# Related memories exhibit interference during learning in adults but later retrieval in children

**DOI:** 10.1371/journal.pone.0356464

**Published:** 2026-09-11

**Authors:** Merron Woodbury, Chunan Duan, Eryk Brol, Rena Patricia Seeger, Tiantian Yang, Margaret L. Schlichting

**Affiliations:** Department of Psychology, University of Toronto, Toronto, Ontario, Canada; University of Utah, UNITED STATES OF AMERICA

## Abstract

To build knowledge, learners of all ages must relate new information to their past experiences. While the interactions among prior and new memories have been extensively studied in adults, they are not yet well characterized in children. There are reasons to expect that children and adults may differ substantially in how related memories interact when memories are first formed as opposed to later referenced, but this has yet to be directly assessed. Here, we had children (4–16 years old) and young adults learn new object locations which either could or could not be incorporated into previously learned two-dimensional maps, i.e., initial memories. Afterwards, they retrieved initial maps from memory. Importantly, initial and new learning occurred on the same day, allowing us to characterize how related spatial memories interact when acquired close in time. We found that during learning, adults but not children were impeded by their recent memories such that they were slower to learn locations within a previously encountered map. However, the opposite developmental pattern emerged at retrieval: Children but not adults demonstrated interference such that they remembered an initial map worse after they had formed new memories within it. Overall, our results suggest that while children and adults may both experience interference among related memories, this interference manifests at different stages: during learning for adults but later memory retrieval for children.

## Introduction

Memories do not exist in isolation but rather interact in complex ways. While navigating the world, children and adults alike frequently face opportunities to update their existing knowledge with new information, such as when learning where a playground is located relative to a new friend’s home on a playdate. Decades of research investigating this process in adults [1–6] has shown that while learning something new, people bring to mind prior related knowledge (e.g., known landmarks), thereby shaping how both new memories are formed [7,8] and old ones are remembered [9]. Little is known about how such memory-memory interactions unfold in younger learners, however, despite strong reason to expect developmental differences. Specifically, past work hints that children may be less susceptible to influence from related memories during learning [10–12] but even more sensitive when memories are later queried [13–15]. Yet, no study to date has directly compared how memory-memory interactions influence behaviour at these different points in the life of a memory. Here, we combined assessments of learning itself and later memory retrieval to characterize how related memories influence one another in distinct ways across development.

While adults can bring to mind their prior memories to shape new learning, the same may not be true for children. In adults, prior knowledge can have either a positive or negative influence on learning, termed proactive facilitation and interference, respectively. New information can be learned faster [1,16,17] and retained longer [18–20] when it can be incorporated into prior knowledge (for reviews see [4,5]). However, prior knowledge can sometimes impede learning, such as when it is weaker [21] or has been acquired more recently [22–24]. Mechanistically, such effects may emerge in part because familiar elements within to-be-learned information cue retrieval of related memories [7,25,26], which can then either facilitate or interfere with new learning. Children by contrast may be relatively less influenced by prior knowledge. For instance, congruency with prior knowledge has an increasingly beneficial effect on memory over development [27–29] (though see [30]). This developmental difference may be explained by both children’s less extensive real-world knowledge as well as their reduced ability to leverage knowledge during learning. With respect to the latter, previous work has indeed found that children do not leverage their existing knowledge to the same extent as adults even when this knowledge is experimentally-induced and approximately matched across age groups [10–12]. In particular, these studies find that when learning related facts or associations, children may be less likely than adults to reactivate [11] and connect [10,12] prior memories with new information. However, while existing work has indexed neurobiological markers (e.g., neural reinstatement [31], looking time [10]) reflecting the extent to which prior memories are leveraged during learning, behavioural consequences have thus far only been measured during a later retrieval period, leaving it unclear whether developmental differences emerge as early as initial acquisition. Here, we asked whether children demonstrate less influence from their previous memories on learning itself, using behavioural measures of acquisition akin to past adult work (e.g., learning accuracy) [16,17].

Related memories can also interact when they are later queried, causing interference such that it is difficult to recall the particular details of each experience. While adults do exhibit such interference [32–34], children may be even more susceptible [13,14,35], perhaps due to immature mechanisms supporting memory retrieval and discrimination [36,37] (e.g., source memory [15,38,39]). Further complicating matters, the degree to which related memories interfere at retrieval may also depend on how they interacted during initial formation. For instance, neuroimaging and behavioural work alike suggests adults may reactivate prior memories during learning in such a way that they emphasize both their differences and similarities from new information, thereby preventing interference among related memories at retrieval [40–43]. By contrast, children may not resolve memory overlap during learning, as discussed above, and thus face exaggerated demands at retrieval. Considering these factors together, we hypothesized that children might exhibit greater interference when retrieving related memories compared to adults.

To assess developmental differences in the interactions among related memories during learning and later retrieval, we had children and adults complete a spatial learning paradigm inspired by influential animal work [44,45]. In a typical experiment, animals learn a map of scent-location pairings over several days, after which they demonstrate facilitated learning of new locations within the familiar map compared to a novel one [44,45]. Work in adult humans [17,19,20] has developed a fruitful analog in which participants acquire two-dimensional (2D) maps of object locations, presented on computer screens or tablets, over days of training and again demonstrate facilitated learning of new locations within a previously learned compared to novel map. Here, we extended upon this work to ask whether spatial memories can support new learning even when formed on the same day, building upon work in associative learning tasks finding encoding can be shaped by memories acquired close in time [21,46–49]. Specifically, adults and children first learned 2D virtual maps of arbitrary object-location pairings after which they learned new locations within an initially learned and completely novel map. We encouraged strong learning of initial maps to increase the likelihood these memories could scaffold new learning by repeatedly exposing participants to these object-location associations during a preceding learning phase until they remembered every association with perfect accuracy. Note that while initial maps were well-learned, we did not expect them to be consolidated prior to new learning. We predicted that even recently acquired initial map memories would facilitate, i.e., speed, new learning in adults [17,19,20] but not children. We also asked whether new memories within a map interfered with how well participants could retrieve initial locations, predicting that children but not adults would exhibit interference and thus worse memory for initial maps when new locations had been introduced.

## Methods

### Participants

Participants were 59 adults (15 male, 43 female, 1 unknown; 17.8–33.3 years old, M = 20.2 years, SD = 2.68) and 161 children (66 male, 94 female, 1 unknown; 3.9–16.5 years, M = 8.2 years, SD = 2.69; most [62%] between 6–9 years old) who were recruited from child-friendly community sites (museum and farmer’s markets) in a large Canadian city (see Supporting Methods in S1 File and Table S1 in S2 File for demographic details and inclusion criteria). Data collection began August 7^th^, 2018, paused due to the COVID-19 pandemic from January 9^th^, 2019 to December 13^th^, 2021, and was completed November 27^th^, 2023. All participants and parents provided permission and written informed consent before the experiment and child participants provided verbal informed assent. Participants were compensated with a small toy (children) or course credit (adults). All procedures were approved by the University of Toronto Research Ethics Board. Analyses were carried out largely as preregistered, with deviations as noted in the methods and results.

### Stimuli & procedure

#### Stimuli.

**Fig 1:**
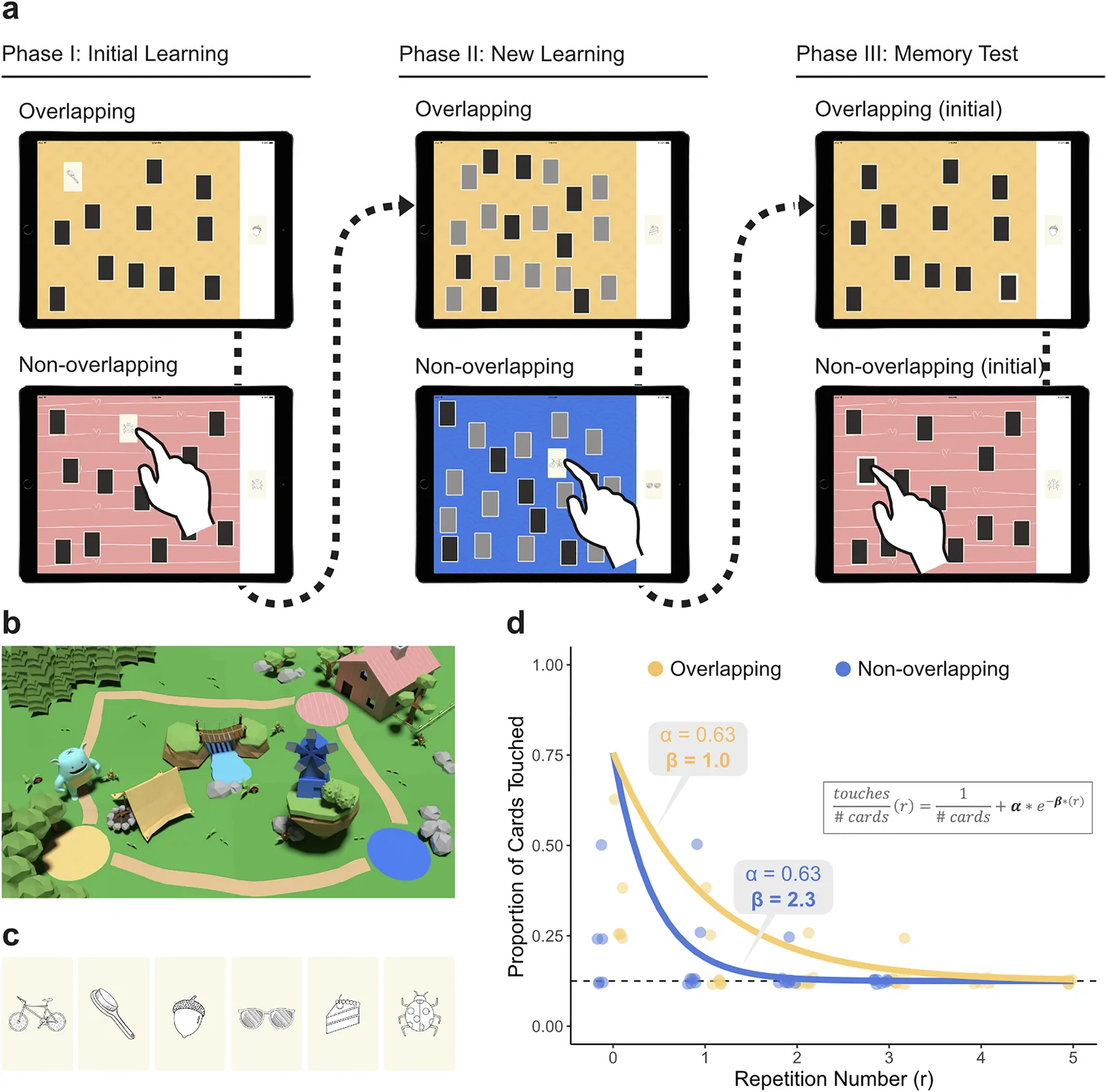
Experimental procedure. **(a)** Example of an adult participant’s progression (dashed arrow) through the learning and memory test phases. Card maps were assigned to distinct colourful backgrounds, i.e., “worlds”. Importantly, a given participant was exposed to a consistent object-location map across Phase I (left) repetitions, with the goal of promoting development of a strong mnemonic scaffold which might support Phase II (middle) learning. Note that map learning order was counterbalanced across participants as described in the text; only some participants experienced this particular order. **(b)** Screenshot of the animation shown between maps where a blue monster walks to the next map’s world, indicated by the yellow, blue, and pink landmarks. **(c)** Schematic examples of objects depicted on face-up cards. Images in the real experiment were colourful illustrations which have been replaced with line drawings of similar but not identical objects in the figure for copyright reasons. **(d)** Data analysis approach showing a representative adult participant’s performance in the new learning phase (blue: non-overlapping; yellow: overlapping) which has been fit with exponential functions. Each dot indicates their performance on a single trial, i.e., proportion of cards they flipped to locate the target object. Black dashed line indicates perfect performance. Annotated with exponential equation used to fit performance as well as resulting learning rates (β) and starting values (α) for each curve.

Stimuli were virtual playing cards distributed face down on coloured backgrounds and presented on an iPad (Fig 1A). Each card had a unique everyday object illustrated on one side (Fig 1C). Participants encountered three different maps of cards, i.e., object-location associations. One map was visited twice through the learning paradigm (“overlapping” condition), once to learn an initial set of cards and then again to learn a new set, while the other two maps were visited only once (“non-overlapping” condition). To make the maps distinct, they were each assigned a unique background (pink, yellow, or blue) and unique spatial organization of cards, the combination of which we call the “world”. Assignment of worlds to conditions was counterbalanced across participants, as was the order in which the overlapping and non-overlapping maps were encountered in the two learning phases (see Supporting Results in S1 File for effects of order on performance). Each phase took a few minutes to complete, with the particular duration depending on participant performance, and participants proceeded almost immediately from one Phase to the next (see Table S2 in S2 File for phase and delay duration details) aside from the time taken to give instructions for the next phase.

#### Phase I: Initial learning.

Participants first acquired memories for two unique card maps in a blocked learning task, only one of which (overlapping) would be revisited in Phase II while the other (non-overlapping) would not. On each trial for a given map, participants were asked to search for the target object presented on the right of the screen by tapping face-down cards to flip them over. Trials were self-paced, and participants could flip as many cards as needed to locate the object, after which a new target would appear. A single learning repetition consisted of successfully locating every object in the map, in random order. Participants completed as many repetitions as necessary until they correctly located every object with a single tap for two consecutive repetitions (learning criterion). Object-location mappings remained the same across repetitions such that participants learned a stable spatial map. To better match task difficulty across age groups, the maps included fewer cards for children (8) than adults (12). After learning one map to criterion, participants proceeded to the next. At the transition between maps, we presented an animation of a friendly monster moving from one to the next, to emphasize that the cards existed in distinct spatial contexts (Fig 1B).

#### Phase II: New learning.

Participants then learned two new sets of object locations, one within a previously encountered map (overlapping) and one in a novel map (non-overlapping). For the overlapping condition, new cards (6 for children; 8 for adults) were added amongst initially learned cards. Initially learned cards were visible to encourage anchoring new object locations within the previously learned map, but they were greyed out and unavailable for selection. For the non-overlapping condition, participants learned the same number of cards in a new map, interspersed with greyed out cards that did not correspond to prior experience.

#### Phase III: Final memory test.

Finally, we gave participants a surprise memory test to assess whether memory for the initially learned locations differed according to whether overlap had been introduced in the map or not. On the memory test for each map, cards were displayed in the same manner as Phase I, with only the initially learned locations (8 for children; 12 for adults) visible. Participants had one attempt to locate each target object (self-paced) and did not received feedback (i.e., cards remained facedown when tapped). To approximately equate the time elapsed between Phase I and Phase III for the two maps, they were tested in the same order they were initially learned.

### Analyses

To first determine whether having initial memories of a spatial map influenced new learning, we compared the rate at which new locations were acquired within the overlapping and non-overlapping maps as follows. Within each phase, we fit a participant’s performance on a given map with an exponential function in which the number of errors they made on a trial, i.e., the proportion of cards that they flipped before finding the target, declined as a function of learning repetition (Fig 1D; Supporting Methods in S1 File). We modeled the proportion of card flips, rather than absolute number as preregistered, to allow direct comparisons between children and adults who had a different number of cards included in each map. Learning rate was operationalized by the parameter indicating how steeply errors declined across repetitions, log transformed.

**Fig 2:**
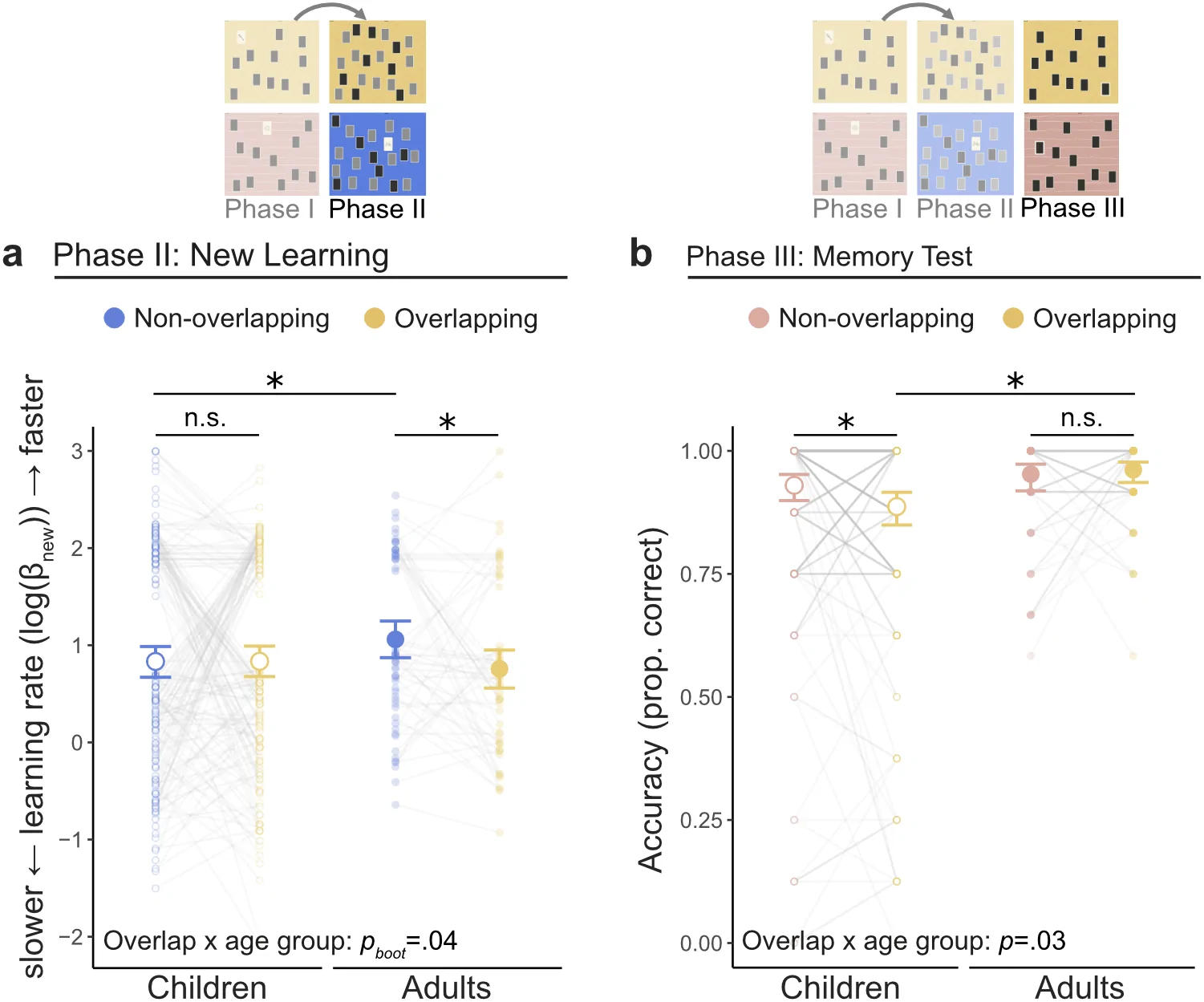
Performance on new learning and memory test phases. **(a)** Learning rate in new learning (Phase II) according to overlap condition and age group. **(b)** Accuracy on final memory test (Phase III) according to overlap condition and age group. Large dots represent model predictions and error bars represent 95% confidence intervals. Smaller dots represent individual participant values. Values from the same participant across conditions are connected with thin gray lines. Significant interactions indicated in bottom left corner of plot. Asterisks indicate significant (p < .05) pairwise differences. Non-significant effects of overlap within an age group denoted as n.s.

We ran linear mixed effects models in R (lmer4::lmer v1.1.25 [50]) to test whether learning rate differed as a function of overlap condition (overlapping or non-overlapping), age group (child or adult), and their interaction. Participant was included as a random effect. Given that learning rates were non-normally distributed (i.e., bimodal; Fig 2A), we ran non-parametric statistics in which we bootstrapped the distributions for each model predictor (lmeresampler::bootstrap v0.2.4 [51]) by repeatedly resampling random participants with replacement and refitting the model (5,000 iterations). We then extracted the following for each predictor: median value, 95% confidence interval, proportion of resamples for which the sign (positive or negative) was the opposite of the median (*p*_boot_). Further, we assessed the significance of each pairwise comparison across predictor levels based on whether the bootstrapped 95% confidence interval for the model prediction at one level included the median value for the prediction at another level.

To test whether performance on the memory test differed according to whether new locations had been introduced to the map (overlapping) or not (non-overlapping), we ran linear mixed effects models in R [50] on memory test performance. We predicted trial-wise accuracy (lme4::glmer; binomial linking function) as a function of overlap condition, age group, and their interaction, including participant-specific intercepts and slopes. We also ran a matching set of models instead predicting log-transformed response times on correct trials (lme4::lmer; Supporting Results in S1 File).

We tested for developmental differences within the child group alone by rerunning all original models comparing younger and older children. Given the uneven sampling of our age range (Fig S1 in S2 File), we used a median split approach to sort children into younger (3.9–7.8 years old, *N* = 80, *M* = 6.2 years, *SD* = 1.01) and older (7.9–16.5 years old, *N* = 81, *M* = 10.2 years, *SD* = 2.28) groups. See Supporting Results in S1 File for consideration of continuous child age.

## Results

### Initial memories influence learning in adults but not children

We first established that there were no differences in how rapidly children and adults learned initial locations within the two first maps (*b* = 0.03, 95% CI [−0.23, 0.31], *p*_boot_ = .4; no differences across conditions; Supporting Results in S1 File) in Phase I, indicating that the task was appropriately matched for difficulty across age groups. We then tested whether children and adults subsequently learned the new locations (Phase II) at different rates within the overlapping compared to non-overlapping map. As predicted, adults were significantly more sensitive to overlap with their prior learning than were children (age group x overlap: *b* = −0.30, 95% CI [−0.65, 0.04], *p*_boot_ = .04; age group: *b* = 0.23, 95% CI [−0.021, 0.47], *p*_boot_ = .03; overlap: *b* = 0.005, 95% CI [−0.23, 0.23], *p*_boot_ = .48; Fig 2A). Children showed no reliable difference in learning rate as a function of overlap (overlapping: median = 0.84, 95% CI [0.68, 0.99]; non-overlapping: median = 0.83, 95% CI [0.67, 0.99]). In contrast, adults were, surprisingly, slower to learn in the overlapping (median = 0.76, 95% CI [0.55, 0.95]) than non-overlapping condition (median = 1.06, 95% CI [0.87, 1.24]). Comparing across age groups revealed that adults were faster than children selectively in the non-overlapping condition, while this developmental advantage was not evident for the overlapping condition. Control analyses (Supporting Results in S1 File) suggested the observed effects were not due to other factors such as rate of initial learning or inclusion of especially fast learners. Together, these results indicate that adults are indeed more influenced by their related memories than children when learning, but in a negative manner consistent with interference.

When examining children alone, older children learned faster than younger in phase II (*b* = 0.31, 95% CI [−.01, 0.63], *p*_boot_ = .03; Fig S1 in S1 File). However, the effect of overlap did not reliably vary across child age groups (*b* = −0.24, 95% CI [−0.70, 0.24], *p*_boot_ = .2). Indeed, learning rate did not significantly differ between the overlapping and non-overlapping condition for either the younger (overlapping: median = 0.80, 95% CI [0.57, 1.03]; non-overlapping: median = 0.68, 95% CI [0.44, 0.91]) or older (overlapping: median = 0.87, 95% CI [0.66, 1.08]; non-overlapping: median = 0.99, 95% CI [0.78, 1.20]) children, though note our ability to detect age differences may have been limited by dense sampling of the relatively narrow 6–9 year old age range. Our developmental effects therefore should be taken to primarily reflect behaviour in middle childhood.

### Interference among overlapping memories at memory test in children but not adults

We next assessed whether memory for initially learned locations, measured on the final memory test (Phase III), differed according to whether new overlapping memories had been formed within the same map. We reasoned that any difference in memory performance across conditions would be due to the introduction of overlap for one map but not the other, given they were both learned to criterion and at similar rates. Overall, the initial locations were remembered with high accuracy by both children (*M* = 0.83, *SD* = 0.22) and adults (*M* = 0.92, *SD* = 0.07). However, children’s memory accuracy was significantly more sensitive to overlap than was adults’ (age group x overlap: *b* = 0.75, *SE* = 0.34, *z* = 2.16, *p* = .03; age group: *b* = 0.42, *SE* = 0.33, *z* = 1.26, *p* = .21; overlap: *b* = −0.53, *SE* = 0.22, *z* = −2.41, *p* = .02; Fig 2B). Children demonstrated less accurate memory for initial locations from the overlapping than non-overlapping map (*z* = 2.41, *p* = .02), while adults demonstrated no reliable difference (*z* = −0.68, *p* = .50). Further, children performed significantly worse than adults selectively on the overlapping (*z* = −3.81, *p* < .001; non-overlapping: *z* = −1.26, *p* = .21) condition. These results are consistent with our predictions that children would be more affected by interference among overlapping memories than adults at the memory test.

Given that adult memory accuracy was at ceiling, we confirmed that the results held when excluding perfect performers (Supporting Results in S1 File). We also found that our results did not substantively change when controlling for the rate at which either the tested locations themselves or new locations from the same overlap condition were learned (Supporting Results in S1 File).

Within children alone, there were no reliable differences in memory test accuracy according to child age group or interactions between child age group and overlap (all *z* < 0.97, *p* > .3). Indeed, worse memory for the overlapping compared to non-overlapping condition, i.e., interference, was exhibited by both younger (*z* = 1.97, *p* = .049) and older (at the trend level, *z* = 1.76, *p* = .08) children. Again, however, the limited age differences within our child sample should be interpreted with caution given the few child participants younger than 6 or older than 9 years old.

### Children and adults demonstrate interference at different points in spatial knowledge building

In an exploratory analysis, we also formally tested whether there were significant developmental differences as to the phase in which interference among overlapping memories was greater, learning (Phase II) or memory test (Phase III). We indeed found that performance (z-scored new learning rate and memory accuracy) differed reliably according to phase, overlap condition, and age group (phase x overlap x age group interaction: *b* = 0.56, 95% CI [0.20, 0.93], *p*_boot_ = .001). Follow-up models within each age group indeed revealed that adults showed significantly more interference during learning than the memory test (phase x overlap: *b* = 0.39, 95% CI [0.11, 0.67], *p*_boot_ = .002). Children showed the opposite pattern numerically, though not reaching statistical significance, whereby interference was greater at the memory test than during learning (phase x overlap: *b* = −0.18, 95% CI [−0.43,.06], *p*_boot_ = .07). Together, these results suggest that there are indeed developmental differences in whether overlapping memories interfere more at learning or memory test.

## Discussion

We set out to test whether related memories formed on the same day interact differently across development during learning and memory retrieval using a spatial learning paradigm. In line with our predictions, adults but not children were influenced by their recent memories of a spatial map during new learning. However, contrary to our expectations, adults were slower to learn new locations which overlapped with a previously learned map compared to those in a novel map, consistent with interference among overlapping memories. In contrast, children showed no reliable difference in learning rate regardless of whether they had previous memories of the map or not. In a sharp distinction from learning, children but not adults showed interference at the memory test, such that children demonstrated worse memory for initially acquired locations when they had formed new memories within the same map. Our results suggest that related memories interfere most strongly at different points in the life of a memory across development: during memory formation in adults but retrieval in children.

Aside from interactions among related memories, we found broad age-related improvements in spatial learning here such that older children learned faster overall than younger children in the new learning phase. This aligns with previous work suggesting that while the building blocks of spatial memory develop early [52,53], there are continued improvements through mid-childhood in precision [54,55], capacity [56], and ability to bind items to locations [57]. As we did not observe similar differences between younger and older children at initial learning, one possibility is that these only emerged when there was greater build-up of non-specific interference from previous learning, which may especially impede younger children [58]. Further, we found that adults were able to achieve similar learning rates to children even while acquiring more locations, contributing to evidence that spatial learning capacity is greater in adulthood.

Adults exhibited interference rather than facilitation during new learning in our study, in contrast to other studies using similar spatial learning paradigms [17,19,20,44]. There are a few key factors that may have led to this difference. One such factor is the timing of initial memory formation relative to new learning. As noted in the Introduction, while most previous studies have established initial maps over days of training, here we had participants acquire them on the same day as new learning. Some theories propose that sufficient time and/or sleep is necessary to consolidate memories before they can effectively scaffold new learning [59–61]. Indeed, shorter lags between initial and new learning may lead to interference [23,24,62] (although see [63]). Thus, it is possible that initial memories interfered with rather than facilitated new memory formation due to their recency. In addition, previous work has found that stronger or more precise prior knowledge increases the likelihood of facilitated learning [21,64,65]. The map memories that adults formed through a single learning session in our task may have been insufficiently strong to ground subsequent learning, in contrast to knowledge established over many days of training in other studies [17,19,20,44]. For example, one mechanistic possibility is that recent memories impede new learning because reactivating and maintaining these memories places large demands on working memory, perhaps especially because they are weaker and not yet consolidated [66,67]. While some previous work has examined how temporal proximity and prior memory strength interact to influence related phenomena, such as the likelihood that initial memories are reactivated and integrated with new learning [48,63,68], future research may test how these factors jointly shape the circumstances under which knowledge facilitates rather than hinders new learning.

Unlike adults, children were significantly less influenced by previous related memories during new learning. We note that because most children in our developmental sample were between 6 and 9 years old, our results should be interpreted as primarily reflecting behaviour in mid-childhood. Our results are consistent with behavioural and neural evidence that children around this age are less likely to access and connect related experiences during learning [10–12]. This may reflect differences in how children approach new learning, as they may be less likely to strategically connect related memories [69]. Further, immature neural circuitry in childhood (e.g., hippocampus and prefrontal cortex [70–72]) may constrain memory reactivation and integration processes during learning. Our findings may seem to contradict prior evidence that children are equally, if not more, affected by proactive interference than adults [14,58,73]. However, previous work has often either focused on interference demonstrated during a memory test (discussed below) rather than during acquisition itself, or interference in working memory from very recently encountered information [58]. Critically, the latter source of interference was unlikely here as our task was designed such that it would be improbable to actively maintain initial memories in mind continuously from initial through to new learning in a map (i.e., due to the length of delays between phases (Table S2 in S2 File) and completion of intervening tasks). Instead, children may have been relatively spared from interference because unlike adults, they did not reactivate their initial memories during new learning.

Unlike learning, children but not adults demonstrated reliable interference due to overlap at the memory test. This finding fits with previous work showing that children exhibit even greater interference among overlapping materials than adults when asked to retrieve them from memory [13,14]. Such differences may in part reflect the prolonged development of strategic retrieval processes [74,75], supported by late-developing prefrontal circuitry in the brain [76–78], including mechanisms for the targeted retrieval of a memory from among overlapping ones [36]. Further, previous works suggests that there are substantial improvements between childhood and adulthood in the ability to discriminate between similar memories by remembering their distinct contexts [13–15,79]. As a result, children here may have experienced greater confusion within a map that was visited twice about what they learned and when. It may seem contradictory to suppose that children are less likely than adults to access related memories during learning but more likely to do so at retrieval; however, accessing related memories during a similar learning experience may require a level of flexible retrieval not yet available to children [80–82], compared to explicitly prompted retrieval. Alternatively, children may have demonstrated poorer memory for initially learned locations within the overlapping map due to memory suppression rather than interference. Children, and perhaps especially adolescents [11,83,84], may be able to suppress related memories during learning itself to prevent interference, and as a consequence render them less accessible for later retrieval. However, such a suppression strategy likely requires the use of additional cognitive resources [85,86] and thus might be expected to slow acquisition, which we did not observe. While our study was largely focused on mid-childhood, future work may target adolescence to investigate whether this age group in particular demonstrates such memory suppression, building upon our results to uncover potential non-linearities in the sources of memory interference at different stages of development.

Directly comparing performance across learning and memory test phases revealed that adults exhibited greater interference during learning than memory retrieval while children showed a statistical trend in the opposite direction (not significant). One possible explanation for this seeming trade-off in interference across phases is that slower learning in adults actually signaled the engagement of mechanisms that resolve overlap with prior memories, by either emphasizing their similarities [41,43,87] or distinctions [41,43,87], in turn preventing interference at retrieval. However, children may not have the opportunity to pre-emptively manage overlap if they do not reactivate related memories during learning, thus placing greater demands on their immature strategic retrieval processes [36,74,75]. If such a trade-off is indeed at work, we would expect a relationship across individuals such that greater interference during learning is associated with less interference at memory test. While we did not find evidence of this correlation in an exploratory analysis here (adults: *p* > .3; children: *p* > .4; Supporting Results in S1 File), future work may empirically test this idea by interrogating individual differences among adults, and how these are linked to factors such as strategy use [49,88,89] or spatial ability [16], in a targeted manner. Moreover, more research will be needed to track whether increases in interference during learning over development coincide with reductions in interference at retrieval, reflecting a shift to more proactive management of memory overlap with age. Further, it remains to be seen whether the limited differences we observed between early childhood and mid-adolescence would bear out with more dense sampling of this age span. Future work may investigate whether there is an inflection point in late adolescence when these signatures rapidly emerge, as may be suggested by our results, or instead a more gradual trajectory signaling incremental improvements in proactive management of memory overlap.

The current study provides evidence for an important developmental shift in how recent and new memories interact. While previous learning interferes with the acquisition of new spatial memories in adulthood, children’s learning is significantly less influenced by their recent memories, perhaps because children are less likely to access their related memories during new learning. However, children are instead more vulnerable to interference from overlap when retrieving initial memories. Together, these results suggest key differences in how children and adults build knowledge through related learning experiences. Our findings pave the way for future research into the mechanisms that produce different forms of memory interference across development as well as age-specific factors that may reduce barriers to learning for children and adults.

## Supporting information

S1 File Supporting Methods and Results. (DOCX)

S2 File Supporting Tables and Figures. (DOCX)
