# Supplementary material for: Related memories exhibit interference during learning in adults but later retrieval in children
Source: PLoS One. 2026 Sep 11;21(9):e0356464. doi: 10.1371/journal.pone.0356464 (PMC13567822; doi:10.1371/journal.pone.0356464)
Supplement: Supplementary file 1 [file pone.0356464.s004.pdf]

Authors' Response

Our sincerest thank you to the Reviewers and Editor for their time and thoughtful consideration of our manuscript. The Reviewers found our manuscript statistically rigorous and the study carefully designed; however, they had a few suggestions for improvement which we have revised the manuscript to address as detailed in this letter. For example, Editor and Reviewer comments prompted us to reassess and adjust our use of Bayesian statistics; better motivate and clarify details of our single-day learning paradigm, include additional detail in our Introduction; and highlight additional outstanding questions regarding strategy use and developmental differences into adolescence in our Discussion. We thank the Reviewers and Editor again for their suggestions which we believe have greatly strengthened the manuscript. Below, Editor and Reviewer comments are in italics and our responses are in normal font.

EDITOR

Thank you for submitting your manuscript to PLOS ONE. After careful consideration, we feel that it has merit but does not fully meet PLOS ONE's publication criteria as it currently stands. Therefore, we invite you to submit a revised version of the manuscript that addresses the points raised during the review process.

The comments from the the reviewers are listed below. I agree with the reviewers that addressing these inquiries would result in an improved manuscript.

> Thank you for your time and thoughtful feedback, as well as the opportunity to submit a revision. We agree that addressing Reviewer feedback has strengthened the manuscript.

In addition to their comments, I kindly request that you address the following issues/questions I had while reading:

a) small detail but the minimum age of the children is sometimes listed as 4 (e.g., abstract) but other times listed as 3 (e.g., when discussing median split; and Figure S1 looks like youngest children are less than 4). Please clarify and be consistent throughout the text.

> Thank you for your careful eye and for noting this error. The actual minimum age in our sample was 3.9 years old, and so when 4 years old was mentioned this was a typo. We have corrected this error throughout the paper (p. 6 and 10).
>
> With respect to Figure S1, we understand the Editor's point that as dots in the originally submitted figure were to the left of the 4 year old tick mark on the x-axis, it appeared as though the age range extended substantially below 4 years. This was merely an artifact of our plotting approach, in which points were "dodged" in ggplot (i.e., offset slightly left and right) to separate the non-overlapping condition in blue/pink from the non-overlapping condition in yellow. Upon reflection and consideration of this comment, we realized that our approach in the original submission of analyzing age as integers could be improved upon by instead computing a more precise age including decimal places. For the purposes of this revision, we have revised our approach and re-made this figure to reflect these more precise ages. In so doing, we have dropped the "dodging"

approach in the plotting such that, related to the Editor's point, now participants' points are exactly aligned with their age on the x-axis. A helpful side effect if this change is that readers can better visualize the within-participant differences (i.e., as now participant ages are more likely to be unique and therefore dots appearing at the same point along the x-axis are likely from the same participant).

We have repeated all analyses using these more precise ages. While this does not have any effect on our primary analyses which considered the whole group of participants irrespective of age, it did affect our reporting of age range, mean, and standard deviation (p. 6) as well as our median split (p. 10; results on p. 11-13). This change did not have a meaningful impact on any of the results or conclusions, and we have updated the particular statistics throughout the manuscript to reflect the results from analyses using precise ages. (e.g., Supporting Results in S1 File p. 2 and 4).

Thank you for bringing this to our attention and prompting this shift in our consideration of age as a more precise value. We believe this change both improves the work overall and helps to clarify how our age range maps onto the graph in Figure S1.

b) I appreciate the use of Bayesian statistics to complement the frequentist stats that served as the primary approach, but was confused as to why these were ONLY done for null results. Bayes Factors can certainly be used to provide the strength of of the evidence in favor of the alternative model (as compared to null) and readers will benefit from having this information. Related, Bayes Factors can be provided for the significant interaction terms that serve as the primary effects reported in this manuscript and readers will benefit from being provided the strength of this evidence. I kindly request a more thorough reporting of these BFs.

Thank you for this thoughtful suggestion for how to strengthen our results reporting and increase the consistency of our statistical approach. This comment prompted us to think more deeply about how our preregistered frequentist analyses and potential Bayesian follow-ups ought to be combined. Having considered extant perspectives on this issue (Dienes, 2024 *JEP: Human perception & performance*; Schreiner & Kunde, 2025 *JEP: General*), we have arrived at the conclusion that it is more appropriate to solely report our planned frequentist statistics rather than additionally include either partial or comprehensive Bayesian testing.

Our reasoning for this decision is as follows: While other research (e.g., Liu et al., 2023, *Cognition*; Qiao et al., 2023, *Current Psychology*; Tsuji & Imaizumi, 2022, *Scientific Reports*) has adopted a similar approach to the one we employed in our initial submission, in which Bayes Factors are reported solely for non-significant frequentists effects in order to support stronger inferences for null effects, recent work argues that this practice can be problematic (Dienes, 2024; Schreiner & Kunde, 2025). Specifically, Dienes (2024) and Schreiner & Kunde (2025) argue that this approach results in unequal treatment of significant and non-significant effects because they are tested with different assumptions and constraints: While significant effects are subject to frequentist assumptions alone, non-significant effects are additionally subject to those associated with Bayesian tests. This can lead to issues. For example, when non-significant frequentist effects are followed up by a Bayesian test with uninformative priors, there may be a bias to yield evidence in favour of the null because such Bayesian tests have a tendency to produce false negatives (Schreiner & Kunde, 2025).

By contrast, the same false negative bias would not affect results that were significant under the frequentist approach, as no Bayesian follow-ups were conducted. Given the risks of this uneven statistical approach, both Schreiner & Kunde (2025) and Dienes (2024) argue that one should use the same inferential system consistently in reporting the results, regardless of whether a frequentist or Bayesian approach is selected.

As for why we have opted to forgo Bayes Factor reporting altogether rather than incorporating Bayesian statistics more thoroughly, we believe it is most appropriate to follow our preregistered frequentist approach which was planned to mirror previous related work in adults (Guo & Yang, 2019; Sommer, 2017; van Buuren et al., 2014). Dual reporting of statistics from multiple inferential systems runs the risk of producing conflicting findings (Dienes, 2024). Instead, the most straightforward and appropriate course of action is to follow the originally planned frequentist analyses. Consequently, we have removed Bayes Factor reporting for null effects wherever previously included in the Results (Fig 1 and p. 10, 11) and Supporting Results (S1 File p. 3) along with the description of these analyses in the Methods (p. 10). Further, as frequentist statistical tests do not allow for the same interpretative strength for null effects as Bayes Factors, we have adjusted our discussion of null effects accordingly throughout the Results and Discussion (e.g., p. 14 and 15).

We thank you again for pointing out the uneven reporting of Bayesian statistics in our results. Your comment led us to consider potential pitfalls of our previous approach, ultimately allowing us better align our statistical reporting with contemporary recommendations for the field.

Review Comments to the Author

REVIEWER #1

The authors investigated whether children and adults differ in how they: (1) learn novel object-location associations embedded into a previously encountered context, and (2) recall the object-location associations initially learned in that context. The results of this behavioral study suggest that, while children learn novel information at a similar rate independently of whether it is presented in a new or previously encountered context, the latter results in a slower learning rate in adults. Conversely, children's memory performance was significantly lower when the retrieved initial object-location associations were presented in a context in which novel overlapping associations had later been encoded. The authors interpret these findings to suggest that adults display interference during learning, while children at retrieval.

The pre-registration of the study and (anticipated) open availability of data are commendable, and the statistical analyses are sound and well-motivated. Nevertheless, I have a few comments.

Thank you for your time and feedback. We address each of your comments below.

R1.1) *My first major comment is about the timing of the experimental phases. The study paradigm is inspired by previous work which demonstrated learning facilitation of new object-location associations when inserted in a schema-compatible framework; however, unlike*

previous work, this paradigm does not allow ample consolidation time between the acquisition of the schema memories and that of the schema-compatible ones. The authors briefly acknowledge this as a limitation in the Discussion, but in my opinion the short delays need to be more clearly pointed out throughout the entire text, and the claims that what is being tested is the effect of "established prior knowledge" on new learning toned down, as it is unclear whether these un-consolidated memories can actually be leveraged as prior knowledge. For example, I would ask to tone down the sentence at lines 113-116: "We ensured that initial map memories were well-established, and thus could serve as a scaffold for new learning, by repeatedly exposing participants to these object-location associations during a preceding learning phase until they remembered every association with perfect accuracy", as I am not convinced that perfect accuracy after a short learning session ensures well-establishment of a memory and its ready access for scaffolding new learning.

Thank you for highlighting this important theoretical and methodological difference between our work and previous related studies. We agree that the reduced time between initial and new learning in our paradigm compared to those used in prior related work (e.g., van Buuren et al., 2014; Sommer, 2017) may have led to interference rather than facilitation in adults in part because initial memories were not consolidated prior to new learning. We included mention of this account in the original Discussion and retain it in the revision (p. 15). Importantly, we wish to clarify that by referring to initially formed memories as "established" we did not mean to imply that they were consolidated. Rather, we were referencing that memories of the initial locations in the overlapping condition were likely relatively strong given participants had learned them perfectly by the end of Phase I. However, we take the Reviewer's point that referring to these memories as "established" may create the impression that these memories *must* be strong, and perhaps even consolidated. We have therefore made changes to the way we describe these initial memories, discussed further below.

We also wish to highlight that there is a substantial body of literature suggesting that even recent, unconsolidated memories can be leveraged during the acquisition of new knowledge. While the schema literature indeed primarily focuses on the influence of remote, consolidated memories, other work has found that even on the same day, manipulations affecting the likelihood of reactivating related memories during subsequent learning can influence how new information is encoded (e.g., Molitor et al., 2021; Richter et al., 2016; Schlichting et al., 2015; Zeithamova et al., 2012) with either positive (Anderson & McCulloch, 1999; Schlichting et al., 2014) or negative (Bramao et al., 2025; Oren & Ash, 2017) effects. For instance, Zeithamova et al. (2012) found that when learning new associations, adults showed neural evidence of reactivating related pairs that had been acquired immediately beforehand. Moreover, the degree to which initial memories were reactivated during learning predicted how strongly new memories were integrated into initial ones. Such online integration is thought to underpin the benefit of prior memories for learning and/or later memory for new, related information (van Kesteren et al., 2012, *Trends in Neurosciences*; Zeithamova et al. 2012, *Frontiers in Human Neuroscience*). Further, some work finds that new learning is integrated with memories formed on the same day to a similar (Fang et al., 2023) or even greater (Zeithamova & Preston, 2017) extent than those formed on a different day. It was this work that motivated our prediction that even on a shorter timescale, adults would access recent memories during new learning and thus demonstrate facilitation. Notably, however, even if learning can be influenced by recently formed (same-day) memories as

seen in prior work, it is nevertheless possible that consolidation could still shift the nature of these memory-memory interactions.

In context of this prior work, our reasons for having participants form initial and new memories within a single session rather than over multiple days were twofold. First, we aimed to empirically test whether facilitation effects observed in prior adult work using spatial learning paradigms (e.g., van Buuren et al., 2014) were also observed within a compressed timeframe, when initial and new memories were formed on the same day. We predicted that adults would call to mind and integrate initially learned spatial maps with new location memories, demonstrating speeded learning in the overlapping compared to non-overlapping condition as a result. Our results instead suggested that adults were impeded by their recently acquired memories. In the Discussion, we speculate as to why this may be the case (p. 15), including consideration of the shorter timeframe and reduced learning exposure to initial maps compared to prior similar work. Second, we had participants complete initial and new learning in a single session to ensure feasibility for collecting a large in-person developmental sample. The short completion time, typically less than 30 minutes, enabled us to collect data at community venues where many potential participants are only available for a short period.

We have revised our manuscript in a number of places (e.g., p. 5 and p. 15) to highlight the difference between this and prior work using similar spatial learning tasks more clearly as well as to explain our reasoning for this design choice. First, we mention the same-day nature of our experiment in our revised Abstract (p. 2) so that this aspect of the design is clear to the reader from the beginning. Second, we have changed the wording of the passage quoted by the Reviewer to remove the potential implication that initial memories *must* scaffold new learning and to rather emphasize that the task was designed with the aim to establish strong enough initial map memories such that they *may* interact with new learning, positively or negatively (p. 5). Third, we have included explicit description of how the timescale of our paradigm differs from those it was inspired by as well as brief justification for why this was done (p. 5), as outlined above. Finally, we have adjusted the wording everywhere that “established” was used in reference to the initial memories to avoid implying that these memories must be strong or consolidated and rather convey the intention of having participants acquire well-learned maps into which new locations might be incorporated (e.g., p 5 and p7).

In short, I would like the authors to:

R1.1a) *1. Point out and motivate their choice of deviating from previous related work by allowing only short delays between experimental phases in the Introduction and/or Methods of the manuscript, instead of leaving this important note only in the Discussion*

Thank you for this suggestion. In the revision, we now include additional clarification regarding the shorter timescale of our spatial learning task compared to prior work in the Abstract and Introduction (p. 2 and p. 5) and justify our design choice according to the logic laid out in our response to the immediately preceding comment, **R1.1**.

R1.1b) *2. Add details in the Methods regarding the length of both the learning phases and the delays between them. How long was each learning phase on average, and what was the variance within each group? Was there a set delay between learning phases I and II, and II and III? If not, what was the average delay?*

Thank you for this suggestion. The exact time spent on each phase varied across participants as it was tied to each individual's performance (e.g., number of repetitions to reach learning criterion, timing of self-paced responses). There was also no set duration for the delays between phases; rather, this depended on the amount of time required for the experimenter to provide instructions for the next phase.

Overall, the duration of each phase and the delays between them were quite brief, with little variability across participants (reported in the newly added **Table S2** in S2 File, also included below for the Reviewer's convenience). The overall slightly longer duration of each phase for adults than children likely reflects that adults had more cards per map. Delays between Phases I and II were typically only a few minutes long and thus, as highlighted by the Reviewer, our results should be interpreted as reflecting how memories interact when formed close in time. As outlined above, this short timescale was an intentional design choice that allowed us to empirically address how recent memories influence new learning.

Table S2. Duration of each experimental phase (Phases I-III) and delays between phases by participant age group.

Age group		*Phase I*	*Phase II*	*Phase III*	*I-II Delay*[2]	*II-III Delay*[2]
Children	*Median (SE)*	5:00 (0:10)[1]	3:24 (0:10)	1:06 (0:01)	0:48 (0:02)	0:06 (0:00.3)
	Range	1:36-13:30	1:36-13:30	0:48-2:12	0:00-3:30	0:00-0:12
Adults	*Median (SE)*	7:30 (0:35)	3:48 (0:09)	1:12 (0:01)	1:12 (0:05)	0:06 (0:00.5)
	Range	3:42-30:12	2:06-8:36	0:54-1:48	0:42-5:12	0:00-0:12

[1]Durations expressed in minutes:seconds format

[2]Duration of delays between completion of phase I and beginning of phase II (I-II Delay) and between completion of phase II and beginning of phase III (II-III Delay). Delays were calculated as the difference between timestamps saved at the end of the previous phase, i.e., immediately after the participant reached the learning criterion for the second map in the phase, and the beginning of the next phase, i.e., the first frame of the animation depicting the monster moving to the next map. Delays of 0 seconds indicate instances in which the participant chose to progress immediately to the next phase and instructions were given while the monster animation played. Monster animations were between 4 and 8 seconds.

Following the Reviewer's line of comments, we suspect they were curious about whether any differences in delay duration between phases might explain differences in performance. We conducted analyses to formally test this question, as discussed in answer to the Reviewer's comment below (**R1.1c**). Additionally, we found that some participants (N=4 children; N=5 adults) had atypically long delays between Phase I and Phase II (greater than the third quartile by 1.5 times the interquartile range), which could conceivably influence interactions among initial and new memories for the overlapping map. To help ensure our results were not driven by this subset of participants with

atypically long delays, we removed these outliers and repeated our main analyses. We focused specifically on the delay between initial learning (phase I) and new learning (phase II) as this was most relevant to the Reviewer's question about how our results may have been influenced by the recency of prior learning relative to new learning. We found that our results remained similar when excluding these individuals, as depicted in **Response Figure 1** below, suggesting the main findings are present among participants who learned the maps at short and relatively consistent delays.

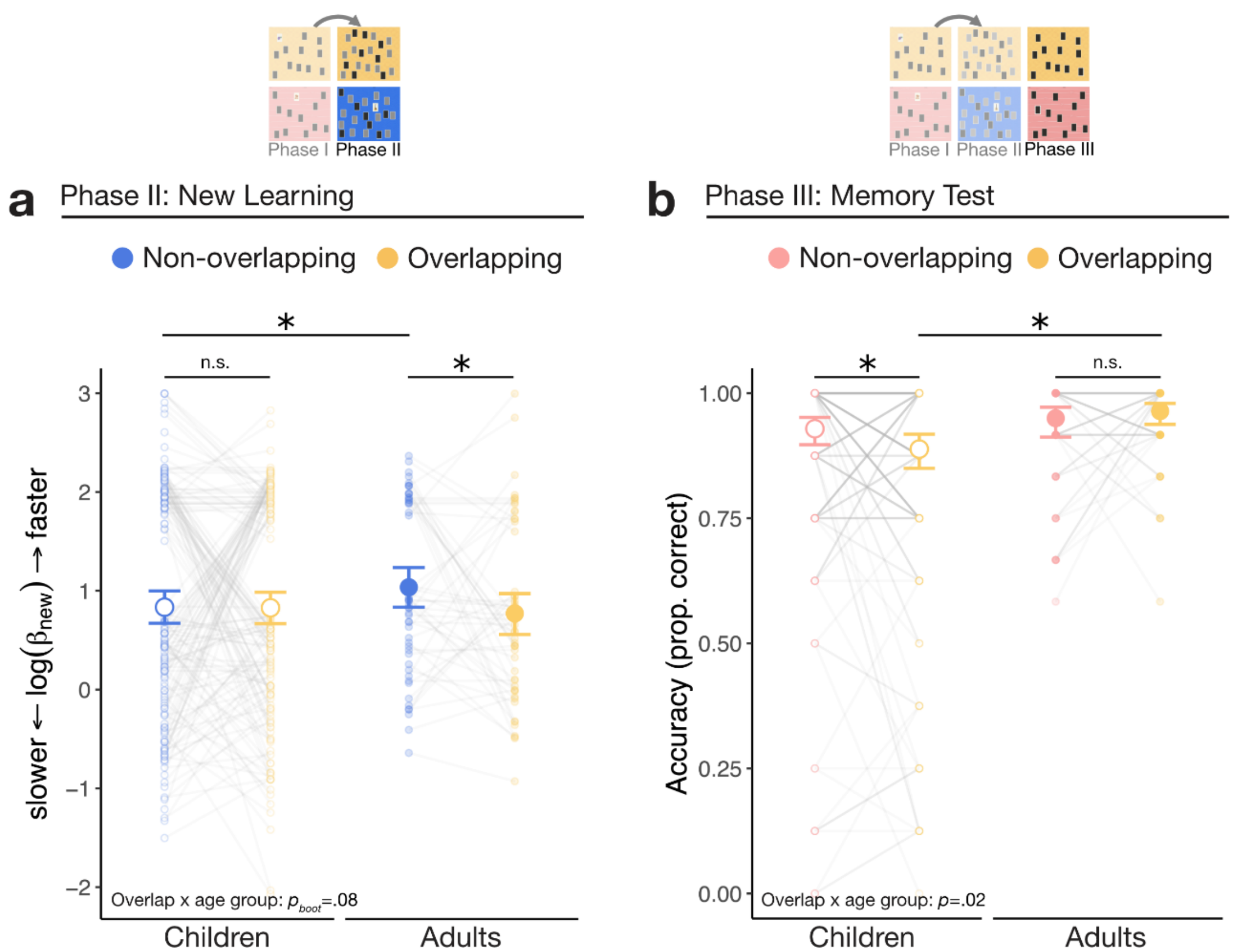

Response Figure 1. Performance on new learning (a) and memory test (b) phases excluding outliers with atypically long delays between Phase I and Phase II learning. (**a**) Learning rate in new learning (Phase II) according to overlap condition and age group. Similar to the pattern in the whole sample, adults were numerically more sensitive to overlap with their prior memories than were children (age group x overlap: *b*=-0.25, 95% CI [-0.63, 0.10], p_{boot}=.08; age group: *b*=0.20, 95% CI[-0.05, 0.45], p_{boot}=.06; overlap: *b*=-0.006, 95% CI [-0.24, 0.22], p_{boot}=.5), though the interaction was no longer statistically significant. Pairwise comparisons within each age group confirmed that as in the complete sample, children showed no reliable difference in learning rate as a function of overlap, while adults were significantly slower to learn in the overlapping than non-overlapping condition. (**b**) Accuracy on final memory test (Phase III) according to overlap condition and age group. As in the whole sample, children's memory accuracy was significantly more sensitive to overlap than was adults' (age group x overlap: *b*=0.85, *SE*=0.36, *z*=2.36, *p*=.02; age group: *b*=-0.50, *SE*=0.22, *z*=-2.23, *p*=.03; overlap: *b*=0.37, *SE*=0.34, *z*=1.08, *p*=.3). Specifically, children still demonstrated less accurate memory for initial locations from the overlapping than non-overlapping map, while adults demonstrated no reliable difference. Large dots represent model predictions and error bars represent 95% confidence intervals. Smaller dots represent individual participant values. Values from the same participant across conditions are

connected with thin gray lines. Significant interactions indicated in bottom left corner of plot. Asterisks indicate significant ($p < .05$) pairwise differences. Non-significant effects of overlap within an age group denoted as n.s.

As there were very few outlier participants with a longer delay between Phase I and II and removing these participants did not meaningfully affect our main results and conclusions, we do not report the results excluding these individuals in the revised manuscript. However, per the Reviewer's suggestion, we now report Phase and delay lengths in the Supporting Information (**Table S2** in S2 File) and clarify in the main text Methods that each phase lasted for a few minutes with very little delay between phases (p.7).

R1.1c) *3. Investigate in an additional analysis whether the length of phase I to phase II delay correlated with subsequent rate of learning in the overlapping environment*

Thank you for this suggested follow-up analysis. We expect the Reviewer is curious about whether participants with longer delays between Phase I and II may show less interference or even facilitated learning in the overlapping maps, consistent with the idea that greater time between learning may allow for memory consolidation to begin and thus allow initial map memories to scaffold rather than impede new location learning. We ran exploratory analyses to assess this possibility with the caveat that we are likely not well-positioned to do so given the limited variability in the delay durations across our participants (**S2 Table**). Further, delays between phases were very short, on the scale of a few seconds to minutes, and thus we suspect it may be unlikely that such small differences in the gap between learning sessions would have substantial consequences for behaviour.

With these limitations in mind, we assessed whether delay duration affected degree of learning interference as per the Reviewer's suggestion by running a linear model in which we predicted a participant's rate of new learning in the overlapping map from the length of delay between phases (in seconds, scaled within age group), excluding those participants identified with outlier delay lengths as discussed above (see response to **R1.1b**). However, rather than longer delays predicting faster learning in the overlapping map, we found a trend (not statistically significant) in the opposite direction whereby longer delays predicted slower learning rates (collapsed across age groups: b=-0.12, 95% CI [-0.32, 0.05], p_{boot}=.09) with no reliable difference across age groups (b=0.09, 95% CI [-0.22, 0.40], p_{boot}=.3). If anything, this finding may suggest that longer delays between initial and new learning may lead to greater interference.

However, the previous analysis does not directly speak to whether length of the delay is related to the degree of interference, as was the Reviewer's original question, and so we also ran a model testing whether the length of the Phase I to II delay predicted the difference in learning rate across conditions (overlapping – non-overlapping) in the new learning phase. If greater delays were to lead to more stable initial memories and thus prevent interference, we would expect a positive relationship such that a longer time period between learning phases would predict less interference or even facilitation (i.e., a condition difference score closer to zero or above zero, respectively). Again, we

excluded the participants with particularly long delays. We found no reliable relationship between the delay and amount of interference in the overlapping relative to non-overlapping map, though numerically (not significantly) longer delays were related to less interference (collapsed across age groups: b=0.09, 95% CI [-0.16, 0.31], p_{boot}=.2) with no reliable differences across age groups (b=-0.14, 95% CI [-0.49, 0.21], p_{boot}=.2). Unlike the previous analyses, here when considering the rate of new learning in the overlapping condition relative to the non-overlapping condition baseline, we find hints that more time following initial memory formation may prevent later interference with learning, though importantly this relationship was not statistically significant.

As mentioned above, the results of these exploratory analyses should be interpreted with caution as our experiment was not well-suited to test the question of how differences in the gap between initial memory formation and new learning might influence memory-memory interactions, given the limited variability and overall short duration of delays. As such, we do not report the results of the above analyses in the paper. However, future work directly manipulating the length of delay may well find that the amount of time elapsed between initial and new learning of related information significantly influences the degree to which learners experience proactive interference. Alternatively, some prior work suggests that even varying delays between a few hours to a few days may not have a substantial influence on how related memories interact (Fang et al., 2023). We leave this question to future studies explicitly designed to test these possibilities.

R1.1d) *4. Discuss alternative explanations for the observed results that do not assume the initially learned map memories were consolidated and able to act as scaffold for the novel ones. For example, is it possible that the learning deficit observed in adults during phase II may reflect taxation of short-term memory capacity during learning, if such learning was being grouped by map (considering there are twice the number of items to encode in the overlapping map)?*

Thank you for raising this point. To clarify, it is not the case that there are twice the number of locations to learn in the overlapping compared to non-overlapping map in the new learning phase. Both maps include the same number of object-location associations eligible for learning in that phase (i.e., cards that are not greyed out in the display). However, we suspect the Reviewer is rather referring to the fact that *across* both learning phases, participants learn a greater number of locations within the overlapping map (e.g., 12 initial and 8 new for adults) compared to the non-overlapping maps such that in the new learning phase, in addition to new locations, additional information is available in memory regarding the greyed out locations in the overlapping map. By contrast, in the non-overlapping map the greyed out locations act as placeholders to mark analogous ineligible locations, but participants have no memory associated with those locations. In the remainder of this response, we address how the additional demand on working memory associated with the ineligible locations in the overlapping compared to non-overlapping map may have shaped learning.

As mentioned in a previous response (**R1.1**), we do not assume that initially learned maps have been consolidated in our task prior to new learning. Rather, we originally predicted that even such recently formed memories could influence, and perhaps facilitate, new learning based on previous literature. In light of our results, we outlined in

the original Discussion (and retained in this revision; p. 15) the possibility that the shortened time between initial and new learning compared to prior similar work may have resulted in interference because initial map memories were insufficiently remote and/or strong to support new learning. To the Reviewer's point, it is possible that this interference was due in part to greater taxation on working memory in the overlapping condition: Adults may have demonstrated slowed learning because they were simultaneously maintaining reactivated, previously learned locations in mind while learning new object-location associations. We agree with the Reviewer that without sufficient time for consolidation or repeated exposure, accessing and maintaining prior related memories may place demands on working memory that slow the learning process. We have added more explicit discussion of this possible explanation for why adults demonstrate interference into the Discussion (p. 15).

Importantly, we suggest it is unlikely that participants actively maintained all learned object-location associations for a map from the time of initial learning through to the new learning phase. Each phase took a few minutes to complete, meaning that for most participants there were several minutes between initially learning a map and encountering new locations within that same map. During that time, it would be unlikely for participants to rehearse the initial locations as they were engaged in learning a new map (see **Fig 1A** for depiction). Consistent with the idea that learning interference was not driven by active, continuous maintenance of initial maps, in an exploratory follow-up we found no evidence for increased interference (slower learning in the overlapping compared to non-overlapping map) when there were fewer intervening maps that could interrupt initial map rehearsal (zero vs one map x overlap: b=-0.16, 95% CI [-0.79, 0.43], p_{boot}=0.3; zero vs two maps x overlap: b=0.21, 95% CI [-0.62, 1.00], p_{boot}=0.3).

In summary, we propose it is unlikely that participants demonstrated interference during new learning merely because they maintained object-locations in mind consistently through both phases, hindering their ability to simultaneously encode new locations. However, as outlined above, we agree with the Reviewer that interference may have arisen from increased taxation of short-term memory due to *reactivation* of previously learned locations from long-term memory, which we now mention in the Discussion (p. 15).

R1.2) *The manuscript is also missing details on a few other points. The introduction is overall clear; however, it is too vague in some places. For example, the paragraph at lines 88-95 states: "existing work has indexed neurobiological markers reflecting the extent to which prior memories are leveraged during learning" – I would like to authors to please explicitly state which markers they are referring to.*

We appreciate this note. We have added more specific details to the Introduction to address this and the immediately following Reviewer comments outlining points they found too vague.

As for the paragraph noted here, the two studies we cite as having quantified whether prior memories are leveraged at learning used measures of neural reinstatement (Schlichting et al., 2022) and eye tracking (Bauer, 2020). Specifically, Schlichting et al.

(2022) used functional magnetic resonance imaging (fMRI) in combination with multivoxel pattern analysis to measure the degree to which related memories where reactivated during learning in children, adolescents, and adults. They found that children (7-10 years old) did not show reliable evidence of reactivating prior related memories during learning in contrast to adults who showed consistent reactivation throughout learning.

Bauer (2020) used an eye tracking approach in which after learning, participants were presented with indirectly related items from overlapping associations (e.g., after learning pairs AB and BC separately, they were presented with items A and C which were indirectly related through shared association with item B) and measured the amount of time spent looking at the indirectly related items compared to unrelated items, under the prediction that only those who had previously reactivated and connected related memories during learning would show preferential looking to the indirectly related items. They found that adults but not children showed this preferential looking towards indirectly related items and further that this preferential looking predicted performance on a subsequent inference task in which they were required to make the link between indirectly related items (e.g., A is indirectly related to C), suggesting the eye tracking measure did reflect greater integration of related memories through learning.

In accordance with the Reviewer's suggestion, we have now included mention of the specific measures used in these studies to quantify prior memory leverage during learning (p. 4). However, we have intentionally kept the descriptions in the main text brief so as not to burden the reader with too much detail which is not necessary to understand the key point. In addition to this particular example noted by the Reviewer as too vague as well as those noted in the following Reviewer comments, we have revised the Introduction elsewhere (e.g., p. 4) to add more details.

R1.3) *Likewise, at line 95: "using behavioural measures of acquisition akin to past adult work" – such as? Please explicitly define these measures.*

Thank you for this comment. Past adult work has measured acquisition by looking at accuracy throughout repeated testing during learning (He et al., 2021; van Buuren et al., 2014), similar to our measure of learning rate. We have revised the sentence highlighted by the Reviewer to explicitly define this measure (p. 4).

And again, at lines 108-111: "[we used] a spatial learning paradigm which was inspired by influential animal work and mirrors approaches that have proven fruitful in human adult research" – brief descriptions of these paradigms are needed, so that the reader can compare them to the one presented in the current study (especially regarding timing, as mentioned above).

The typical paradigm, with slight variations across studies, comprises an initial learning phase in which participants are repeatedly exposed to an arbitrary map of object (human) or scent (rodent) locations. These maps are typically two dimensional in human studies and presented on a screen. As the Reviewer has highlighted, participants typically learn these initial maps through multiple days of study before a new set of

locations is introduced into the maps for participants to learn. Our paradigm largely follows the same format as this prior work with the exception that initial map learning is conducted on the same day as new location learning. We discuss above how this type of same-day approach is not without precedent in the adult literature using non-spatial learning paradigms to study how related memories interact (**R1.1**) and which inspired our adult predictions.

Following the Reviewer's suggestion, in this revision we now incorporate descriptions of the commonalities across our paradigm and prior approaches and highlight the divergence in the timescale of our task compared to prior similar work in the Introduction (p. 5).

R1.4) *Finally, tying into my earlier comment to please specify the "neurobiological markers reflecting the extent to which prior memories are leveraged during learning", I would appreciate some more speculation in the Discussion as to what the behavioral results of this study may imply regarding the differences in neurobiological mechanisms underlying memory-memory interactions in children vs adults (currently very briefly touched upon at lines 346-348).*

Thank you for the feedback. We appreciate the suggestion and have added in additional discussion of the potential neural mechanisms supporting improvements in the ability to resolve competition at memory retrieval (p. 16). We reason such differences may underlie, at least in part, the developmental differences observed here at memory test. However, given the purely behavioural nature of our findings, we are not comfortable speculating further about specific neurobiological mechanisms. While our results do suggest that there are differences in how related memories interact with one another across development – both during learning and later memory retrieval – they cannot speak directly to differences in the brain underpinning these behavioural effects. We hope the Reviewer will find this revision in our Discussion helpful while also understanding our hesitation to speak too far beyond our data.

Minor comments:

R1.5) *Line 72 – the term "initial memories" is somewhat unclear; consider using "initially learned" (also in the rest of the manuscript)*

Thank you for pointing this out. We have adjusted the wording to refer to "related" rather than "initial and new" memories in that sentence (p. 3), as we agree the phrasing on this line in our original submission was unclear. We opted to use "related memories" rather than "initially learned" to ideally avoid confusion on the reader's part about what "initial" was relative to in this context and to highlight that we were specifically interested in how memories for connected or overlapping information interact. We have also adjusted the wording in a number of other places where our use of the term "initial memories" may have been unclear or confusing for similar reasons (e.g., p. 9 and p. 11).

R1.6) *Line 74 – The phrasing of "while adults can bring to mind their prior memories such that it shapes new learning" is awkward, I would suggest simplifying to: "while adults can bring to mind their prior memories to shape new learning"*

Thank you for this; we have adopted the Reviewer's suggested phrasing (p. 3).

R1.7) *Line 76 – please insert a comma before "respectively"*

Thank you for pointing out this mistake. We have fixed it (p. 3).

R1.8) *Line 79 – the sentence structure suggests that the studies referenced here were all conducted in adult humans, but a work conducted in rodents is cited. Please rephrase to make this clear.*

We appreciate this note. We have adjusted the wording of this sentence to not imply that all of the cited work was done in adult humans (p. 3).

R1.9) *Line 81 – for consistency with earlier sections of the Introduction, I suggest using "facilitate" rather than "ground"*

Thank you for pointing this out. We have changed this wording according to the Reviewer's suggestion (p. 3).

R1.10) *Line 97 – I find "to recall what happened where and when" too colloquial, I suggest using "to recall contextual details". Please similarly update line 363.*

Thank you for the suggestion. We have adjusted the wording in those sentences (p4 and p17) to achieve a more formal tone.

R1.11) *Line 228 – I would add "subsequent" learning*

Thank you for the suggestion. We have done so (p11).

R1.12) *For consistency with the Methods, in the Results please explicitly refer to the learning phases corresponding to each presented test (e.g., lines 229-232: phase I; lines 232-236, etc.).*

Thank you for pointing out this discrepancy in terminology between the Methods and Results. We have added in mentions of the experimental phase throughout the results reporting (p10-13) to improve clarity.

R1.12) *Line 243 – please change "confirmed" to "suggested"*
Line 282 – please change again "confirmed our results…" to "investigated whether our results…"

Thank you for these suggestions. We have adjusted the wording of both sentences accordingly.

R1.13) *Lines 297-299 – in this otherwise very solid paper statistics-wise, I would prefer not to see results reported as "marginally significant". Please change this to (something along the lines of): "Children tended to display higher interference during memory test, although this difference was not significant". Please also add the caveat that the difference was not significant to line 377 in the Discussion.*

Thank you for pointing this out. We have adjusted the wording in both places (p13 and p16) to emphasize that this interaction was not statistically significant.

R1.14) *Lines 395-397 – as this experiment is purely behavioural, stating that "we demonstrate key differences in how children and adults build knowledge" is, in my opinion, too strong a claim. Change to (something along the lines of): "these results suggest there may be key differences in how children and adults build knowledge"*

We appreciate the feedback. We have adjusted the wording of this sentence accordingly (p. 18).

R1.15) *Supplementary Methods – please put the list of ethnicities in a table instead of text, and in descending order from most to least represented ethnicity.*

Thank you for the suggestion. We have replaced the paragraph reporting participant ethnicity with a table (**Supporting Methods** in S1 File, p1, Table S1 in S2 File) including this information.

R1.16) *Supplement – page 5, typo: "there was still in interaction"*

Thank you for pointing out this error. We have corrected it (**Supporting Results** in S1 File, p. 5).

Thank you again to Reviewer #1 for their helpful feedback and suggestions for improving the paper.

REVIEWER #2

This manuscript describes a behavioral experiment with adults (n=59) and children (n=161, 4-16 years) that investigates interactions among related memories during learning and retrieval. Participants memorized the identities of each card within different maps. One map background was repeated to create potential interference. The number of cards within the map was larger for adults compared to child to equate difficulty. Learning rates were the same for children and adults when learning the non-overlapping maps. However, adults' learning was more sensitive to the overlap manipulation than children's: adults learned the overlapping map more slowly than the non-overlapping maps, whereas children showed equivalent learning rates in both conditions. By contrast, for memory retrieval, children's memory was more sensitive to overlap than adults. Children's memory accuracy was lower for the overlapping map compared to the

non-overlapping map, but adults' memory did not differ between the maps. Overall, I enjoyed reading this manuscript. I think the experimental design is a clever and appropriate way of targeting the research questions, the writing is clear, and figures provide useful visual aids. I have only a few questions about interpreting the results:

Thank you for your consideration and feedback. We are glad you enjoyed reading our paper! We have addressed each of your questions below.

R2.1) *1) Given the age-group differences between children and adults regarding the overlap manipulation for learning and retrieval and the large age range tested in children, it is somewhat surprising that the effect of overlap did not vary as a function of age within children. How do you interpret these seemingly conflicting findings? Do you think it is an issue of statistical power within the child group given the dense sampling between 6-9 years? Potentially there is an inflection point after age 16 rather than continuous change?*

Thank you for this comment. We agree that the minimal age differences observed within our child sample are somewhat surprising. Notably, inspired by the Editor's question regarding our age range, we repeated our analyses of child age (median split and continuous) using more precise participant ages and still found the same limited differences according to child age (main text p. 11 and p. 13, **Fig S1** in S2 File**,** Supporting Information in S1 File p. 4). However, as the Reviewer mentions and as we highlight in the Results (p. 12), the dense sampling of 6-9 year olds relative to other child ages likely reduced our statistical power to detect such developmental differences within the child sample. Therefore, we believe our results should be interpreted mainly as reflecting behaviour during middle childhood. We have revised the Results (p12 and p13) and Discussion (p. 15) to further highlight this important caveat when interpreting our results.

Nevertheless, the Reviewer raises an interesting point that rather than continuous developmental change, there may be non-linear effects such that at a certain point in adolescence behaviour shifts to resemble adult performance. Alternatively, adolescents may behave differently altogether from either younger children or adults. This would make an intriguing line of future investigation, and we have added mention of this open question regarding the adolescent period in the Discussion (p. 17 and p.18).

R2.2) *2) Figure 2A seems to show a striking bimodal distribution of learning rates in both children and adults, where there is a larger cluster of slower learners and a smaller but still large cluster of much faster learners. How do you interpret these different clusters in terms of potentially different learning strategies? I see there is an exploratory analysis relating interference during learning and interference during memory, which found no relation, but do these different clusters of learning speed relate to later memory performance?*

We appreciate this point. The Reviewer is correct that there is a clear bimodal distribution of learning rates, which emerged due to the nature of modeling an exponential learning curve (**Fig 1d**) on performance with both a discrete x-axis (learning repetitions) and y-axis (proportion of errors per trial). Because x-values for the learning curve were discrete integers and y-values were discrete proportions with only as many

possible values as there were cards in a map (e.g., 12 for adults and 8 for children in the initial learning phase), certain learning rates were impossible to fit to these values. Learning rates in the upper distribution all reflect instances in which the participant reached the learning criterion in the minimal number of repetitions possible (i.e., three). In these cases, variability in the learning curve slope was entirely dependent on the number of errors made in the initial repetition after which the participant performed perfectly. Learning rates in the lower distribution were all instances in which the participant learned in more than the minimum number of repetitions (i.e., four or more). The slight difference in where this learning rate gap was located between age groups was due to the different number of cards per map across groups, and thus different discrete numbers of possible values for proportion of errors made per trial.

In our original submission, and also retained in this revision, we took a number of steps to ensure the bimodal nature of the distribution did not have unanticipated effects on our results, which we outline here for clarity. First, we used nonparametric tests to account for the non-normal nature of the data: Specifically, we used a bootstrap approach to generate distributions for each model predictor rather than relying on parametric tests which assume normality. Second, we ran control analyses to confirm that our results held when excluding the upper distribution of fast learners in each condition (Supporting Results in S1 File p4-5). Indeed, we found that even when excluding this upper distribution, adults still showed slower learning on the overlapping compared to non-overlapping condition, suggesting the interference effect was not an artifact of the bimodal learning rate distribution.

Nevertheless, we appreciate the Reviewer's suggestion that the bimodal distributions may reflect different learning strategies, as it is possible that those within the upper distribution who learned in the fewest number of repetitions employed a particularly effective strategy. While we believe our data are not particularly well-poised to uncover differences in strategy use between the groups, we performed several exploratory analyses inspired by the Reviewer's comment to characterize potential differences between these two groups. First, we aimed to test whether fast and slow learner groups reflected consistent individual differences in strategy use such that the same participants were "fast" learners across every map in the entire task. To test this idea, we labeled participants according to whether they were fast or slow learners (i.e., in the upper or lower portion of the bimodal distribution) on each of the four maps (initial and new overlapping, initial and new non-overlapping). We found that it was relatively rare for a participant to be a fast learner across all four maps (11.9% adults and 0% children). This infrequency suggests the upper portion of the learning rate distributions is not comprised of the same set of faster learning participants across all phases of the task, but rather different participants fall within the fast or slow portions across different parts of the task. Additionally, we did not find that learning group (fast or slow) predicted memory accuracy for initial maps, either collapsing across age group (overlapping map: b=-.06, SE=0.18, z=-0.33, p=.7; non-overlapping: b=-0.17, SE=0.18, z=-0.94, p=.4) or interacting with age group (overlapping: b=0.04, SE=0.18, z=0.24, p=.8; non-overlapping: b=0.02, SE=0.18, z=0.14, p=.9). Together, our results do not clearly point to consistent individual differences in strategy use which give rise to fast or slow learning, and as a result differences in memory performance. However, we did not systematically record participants' subjective reports on strategy use, as we prioritized quick data collection to increase feasibility at our community venues.

Delving into the bimodal distribution of learning rates further, additional analyses did not reveal any hints that whether participants fell within the fast or slow learner group was dependent on experimental circumstances. We asked whether participants acquired the maps particularly fast under specific experimental conditions, such as when a map was learned first vs. second or in one of the three "worlds" (yellow, pink, or blue). However, we found no effect of map order on how likely participants were to be categorized as a slow or fast learner in either phase (logistic regression; phase I: b=0.14, SE=0.11, z=1.30, p=.2); phase II: b=-0.10, SE=0.10, z=-1.0, p=.3), nor was there an effect of the world (phase I: yellow vs blue [b=-.008, SE=0.15, z=-0.5, p=.96], pink vs blue [b=-0.05, SE=0.15, z=-0.33, p=.7]; phase II: yellow vs blue [b=0.06, SE=0.14, z=0.47, p=.6], pink vs blue [b=0.14, SE=0.14, z=0.95, p=0.3]). Together, these exploratory analyses did not reveal reliable experimental conditions that gave rise to particularly fast compared to slow map learning.

In summary, we did not clearly identify either a group of participants who consistently demonstrated fast learning or a particular set of experimental circumstances that lead to faster learning. However, future work may more systematically explore differences in strategy use and/or individual differences in spatial abilities that may lead to rapid acquisition of such two-dimensional maps and further, differences in how related spatial memories interact with one another. We have added a call for such research in the revised Discussion (p. 18).

Thank you to Reviewer #2 for their time and thoughtful questions.
