# Supplementary material for: Related memories exhibit interference during learning in adults but later retrieval in children
Source: PLoS One. 2026 Sep 11;21(9):e0356464. doi: 10.1371/journal.pone.0356464 (PMC13567822; doi:10.1371/journal.pone.0356464)
Supplement: Supplementary file 2 [file pone.0356464.s001.docx]

## Supporting Methods

### Participant details and exclusions

Beyond the participants included in the main analyses (N=59 adults, N=161 children), an additional eight adults and 93 children participated but were excluded because they did not complete all experimental phases (adults: N=8; children: N=79), there was a technical error with the iPad task (children: N=3), they did not speak fluent English (children: N=5), parents did not provide the child’s age (children: N=1), or a participant had a diagnosed mental illness or neurological disorder (children: N=5). One additional adult was excluded from analyses due to a data issue (multiple responses recorded per trial) discovered after data collection had concluded, leaving one less in the sample than we had preregistered (N=59). Children’s incompletion rates were higher than might be expected for traditional laboratory testing given that testing was completed at public community venues. Further, the common demographics at our testing sites (museum and farmer’s markets) meant that most (62%) children included were between 6-9 years old, though we tested any interested child between 3-16 years old (see **S1 Fig** for histograms of child age distribution).

Data collection occurred in the city of Toronto, Canada, and as such most participants resided in urban areas in this diverse, multicultural city. Self-reported ethnicity for included participants is summarized in **Supplementary Table 1**. Some participants or parents chose not to complete the demographics survey (N=2 adults; N=41 children) and as such their ethnicity is reported here as “Chose not to specify” and their age was included in analyses as age in years as an integer, reported directly at the time of data collection, rather than calculated from date of birth. We did not ask participants for information regarding their socioeconomic status, but note that Toronto as an urban centre has both a high cost of living and high median household income ($84,000 Canadian dollars; Canadian Census 2021) relative to other parts of Canada.

### Learning rate estimation

To measure the rate at which participants learned locations within each map in each learning phase, we fit their performance with the following exponential function:

$$\frac{touches}{\# cards}\left( r \right)=\frac{1}{\# cards}+\alpha*e^{-\boldsymbol{\beta}*(r)}$$

where $\frac{touches}{\# cards}$ is the proportion of cards within a map they flipped before locating the target object on each trial. Proportion of cards touched is predicted as a function of learning repetition number ($r$) and decreases as a participant learns the object locations until they reach perfect performance ( $\frac{1}{\# cards}$ ) on two consecutive learning repetitions. We used the proportion of cards rather than the raw number of cards touched, as originally preregistered, so that we could compare model fitting results for children and adults who learned different numbers of cards. We operationalized learning rate as the log-transformed beta ($\beta$) parameter which indicates how steeply number of cards touched decreased over learning. This learning rate measure allowed us to be sensitive to gradual learning-related changes in error behaviour. We fit the y-intercept of the learning curve ($\alpha$) for performance across an entire learning phase, rather than for each map within a phase, to ensure that only learning rate ($\beta$) varied between compared conditions.

## Supporting Results

### Initial learning is similar across age groups and conditions

As noted in the main text, there were no reliable differences across age groups in learning rate during the initial learning phase across maps (*b*=0.03, 95% CI [-0.25, 0.31], *p_boot_*=.4). We confirmed that there were still no significant age differences when separately considering both the map that would later have new locations introduced, i.e., overlapping condition (children: median=0.78, 95% CI [0.56, 0.99]; adults: median=0.68, 95% CI [0.54, 0.82]), and the one that would not, i.e., non-overlapping condition (children: median=0.61, 95% CI [0.46, 0.77]; adults: median=0.65, 95% CI [0.42, 0.87]). Nor were there reliable differences in how quickly these initial maps were learned, either across age groups (*b*=0.07, 95% CI [-0.15, 0.28], *p_boot_*=.3) or interacting with age group (*b*=0.07, 95% CI [-0.32, 0.45], *p_boot_*=.4). Further, within the children alone, there were no reliable differences according to child age group (*b*=0.12, 95% CI [-0.20, 0.45], *p_boot_*=.2) or overlap condition (*b*=0.08, 95% CI [-0.27, 0.42], *p_boot_*=.3). Nor was there a significant interaction between child age group and overlap *(b*=-0.02, 95% CI [-0.46, 0.41], *p_boot_*=.5*).* Similar learning rates across our sample indicate that our paradigm successfully minimized broader developmental differences in performance. Further, we confirmed there were no significant differences in learning between the initial maps prior to introducing overlapping information.

### Effects of overlap on memory test response times

For our primary analyses on the memory test, we considered the accuracy with which participants remembered initially learned locations. As preregistered, we additionally considered response times on correct trials. We reasoned that response time may be a more sensitive and appropriate measure for assessing overlap effects within especially the high-performing adult group. As participants were often much slower to respond on the first trial of each test while they became oriented to the task, we excluded these trials from response time analyses. Examining the remaining response times on correct trials revealed a significant difference across age groups (*b*=-0.11, *SE*=0.026, *t*(168.6)=-4.43, *p<*.001), such that adults were overall faster to respond than children (overlapping: *z*=5.05, *p<*.001; non-overlapping: *z*=4.43, *p*<.001). Further and in contrast to our primary results with accuracy, there was no interaction between overlap and age group (*t*(140.3)=-1.00, *p*=.32) for response times. Instead, there was a trend towards a main effect of overlap (with overlapping being marginally slower than non-overlapping) across age groups (*b*=0.031, *SE*=0.017, *t*(222.4)=1.85, *p*=.065). For completeness, we also performed follow-up exploratory analyses of response time within children and adults separately, the results of which were broadly consistent with our main findings on accuracy: Adults’ speed was not reliably affected by overlap (*b*=0.002, *SE*=.002, *t*(115.6)=0.13, *p*=.9), whereas children were significantly slower to make overlapping than non-overlapping correct test responses (*b*=0.06, *SE*=0.025, *t*(132.5)=2.32, *p=*.02), suggestive of interference. Within the child group, older children were faster than younger children (*b*=-.075, *SE*=.03, *t*(145.3)=-2.47, *p*=.02), but child age group did not reliably interact with overlap effects (*b*=-.04, *SE*=.04, *t*(132.6)=-1.10, *p*=.3).

### Exploratory analysis of trade-off between learning and memory interference

We conducted an additional exploratory analysis to test whether there was a trade-off in interference effects across participants such that those demonstrating greater interference during learning showed less interference at memory retrieval. Such a result might indicate that slowed learning in the presence of initial memories in fact reflects an adaptive process which prevents interference among related memories at retrieval. For each participant we calculated a z-scored difference score reflecting the degree to which they experienced interference during new learning (non-overlapping – overlapping learning rate) and tested whether this was a significant predictor of interference on the memory test (non-overlapping – overlapping initial map accuracy, z-scored) using linear models (stats::lm). We found no reliable relationship between the degree of learning and memory interference across age groups or interacting with age group (all *t*(216)<1.0, *p>*.3). Further, this relationship was not significant within either adults (*t*(57)*=*0.80, *p=*.4) or children (*t*(159) *=*0.93, *p=*.4) when running separate models for each age group. The same was true when considering memory test response times on correct trials rather than accuracy (full sample: all *t*(210)*<*1.2, *p>*.2; adults: *t*(57)=-0.33, *p*=.7; children: *t*(153)=.09, *p*=.9).

### Effects of continuous child age

In addition to considering the effects of child age when splitting children into younger (3.9-7.7 years old) and older groups (7.8-16.5 years old), we also conducted all main analyses considering the influence of continuous child age in years, as preregistered. We found the same pattern of results as when comparing median-split child age groups. Specifically, in the initial learning phase, there was no significant effect of continuous child age on learning rate across maps (*b*=.08, 95% CI [-.06, 0.22], *p_boot_*=.2) nor an interaction with overlap condition (*b*=-.02, 95% CI [-0.21, 0.18], *p_boot_*=.4) or main effect of overlap (*b*=.06, 95% CI [-0.15, 0.27], *p_boot_*=.3).

New learning rate showed a significant difference according to child age such that older children learned faster than younger across conditions (*b*=0.14, 95% CI [-.02, 0.31], *p_boot_*=.04) but child age did not interact with overlap condition (*b*=-.07, 95% CI [-0.31, 0.16], *p_boot_*=.3) nor was there a significant effect of overlap on learning rate (*b*=.001, 95% CI [-0.22, 0.22], *p_boot_*=.5), just as when considering child age groups.

Finally, on the memory test, there was no reliable influence of child age on accuracy across conditions (*b*=-0.24, *SE*=0.19, *z*=-1.26, *p*=.2) but children did demonstrate less accurate memory for the overlapping compared to non-overlapping condition (*b*=-0.57, *SE*=0.24, *z*=-2.32, *p*=.02) in a way that did not differ significantly according to child age (*b*=.06, *SE*=0.18, *z*=0.33, *p*=.7). Again, considering the effects of continuous child age produced the same pattern of results as child age groups.

### Control analyses

### Effects of overlap on new learning remain when accounting for other potential factors

To verify that the effects of overlap on new learning rate could not be explained by other factors we conducted several control analyses. We first confirmed that the adult interference effect, whereby learning was slower on the overlapping compared to non-overlapping map, remained significant when excluding the fastest learners from each condition (N_overlapping_=43 and N_non-overlapping_=37 included; *b*=-0.17, 95% CI [-0.36, .02], *p*_boot_=.04), i.e., the upper portion of bimodal learning rate distributions (learning rate ≥ 3). In addition to the nonparametric statistical approach presented in the main text, results from this control analysis indicate that the interference effect was not an artifact of bimodal learning rate distributions.

We additionally tested whether the pattern of results for new learning rate in both children and adults remained when controlling for individual learning rates on the corresponding initial map (overlapping or non-overlapping). Indeed, there was still an interaction between age group and overlap when including initial learning rate as a nuisance predictor (*b*=-0.30, 95% CI [-0.64, .04], *p*_boot_=.04) such that adults demonstrated slower new learning for the overlapping (median=0.76 , 95% CI [0.55, 0.95]) than non-overlapping (median=1.06 , 95% CI [0.87, 1.24]) map and children showed no such effect (overlapping: median=0.84, 95% CI [0.68, 0.99]; non-overlapping: median=0.83, 95% CI [0.67, 0.99]). Further, initial learning rate did not significantly predict new learning rate for either age group (adults: *b*=.05, 95% CI [-0.14, 0.23], *p*_boot_=.31; though children showed a trend: *b*=-0.10, 95% CI [-0.25, .04], *p*_boot_=.08). In sum, the rate at which initial locations were acquired did not have a reliable influence on how quickly the corresponding overlapping (or non-overlapping) locations were learned in the subsequent learning phase.

### Effects of overlap on memory performance remain when accounting for other potential factors

Given potential ceiling effects on the memory test due to excellent overall performance, we asked whether the same results held when excluding those who performed perfectly (100% accuracy) on both tests. We indeed found that even with this restricted sample (*N*=45 adults and *N*=115 children) the interaction between age group and overlap remained significant (overlap × age group: *b*=0.74, *SE*=0.34, *z*=2.18, *p*=.03; overlap: *b*=-0.47, *SE*=0.19, *z*=-2.52, *p*=.01; age group: *b*=0.64, *SE*=0.29, *z*=2.19, *p*=.03). Just as within the full sample, accuracy on the memory test was worse for the overlapping compared to non-overlapping condition for children (*z=*2.52, *p=*.01) but not adults (*z=*-0.89, *p=*.4). With the best performers excluded, there were also age differences for both conditions, such that adults were more accurate than children on both overlapping (*z=*-5.15, *p<*.001) and non-overlapping (*z=*-2.19, *p=*.03) conditions. Overall, this analysis confirms that children but not adults demonstrated interference at the memory test.

We tested whether the differing effects of overlap on memory test performance for initially learned maps across children and adults held when controlling for the initial rate at which the tested locations were learned or rate at which the new locations from the same condition (overlapping or non-overlapping) were learned. We found that the interaction between age group and overlap on memory accuracy held as significant when controlling for each (initial learning rate: *b=*0.75, *SE*=0.34, *z*=2.21, *p*=.03; new learning rate: *b*=0.76, *SE*=0.34, *z*=2.25, *p*=.03), such that children demonstrated worse memory for the overlapping than non-overlapping map (initial learning rate: *z*=2.37, *p*=.02; new learning rate: *z*=2.41, *p*=.02) and adults showed no such effect (initial learning rate: *z*=-0.72, *p*=.5; new learning rate: *z*=-0.73, *p*=.5). Further, neither learning rate had a significant influence on memory accuracy for either children (initial learning rate: *b=*0.22, *SE*=0.35, *z*=0.64, *p*=.5; new learning rate: *b=*-0.05, *SE*=0.14, *z*=-0.37, *p*=.7) or adults (initial learning rate: *b=*,-0.053 *SE*=0.12, *z*=-0.45, *p*=.7; new learning rate: *b=*.09, *SE*=0.11, *z*=0.82, *p*=.4).

### Order effects on learning and memory performance

In addition to the previously outlined control analyses, we also tested whether there were differences in how maps were learned and remembered according to the order in which they were encountered. For the initial learning phase, we found no effect of learning order (first or second) on learning rate across age groups (*b*=-0.024, 95% CI [-0.24, 0.19], *p*_boot_*=*.40), though there was a marginal interaction with age group (*b*=0.31, 95% CI [-0.07, 0.69], *p*_boot_*=*.055). Adults learned significantly faster on the second than first map (*b*=0.28, 95% CI [-0.03, 0.59], *p*_boot_*=*.03) while children did not (*b*=-0.024, 95% CI [-0.24, 0.19], *p*_boot_*=*.4). Adults might have benefitted somewhat from practice with the task while children did not show such an effect. When following up on potential order effects during the new learning phase, we found no differences in learning rate according to order across age groups or interacting with age group (all *p*_boot_>.2).

Participants did show an effect of testing order on memory performance. Specifically, participants were more accurate on the second compared to the first memory test across age groups (*b*=0.74, *SE*=0.22, *z=*3.42, *p<*.001; age group × order interaction: *b*=0.52, *SE*=0.34, *z=*1.52, *p=*.13). It should be noted that the order in which layouts were encountered was the same in Phase I and Phase III so that the second map tested was also always the second map learned in the initial learning phase. This order effect may therefore reflect improvement in either the learning or memory task performance with experience. Importantly, we confirmed that controlling for the effect of testing order did not alter our findings as to the influence of overlap on memory performance. Namely, children still demonstrated greater interference than adults (age group × overlap: *b*=0.71, *SE*=0.33, *z=*2.18, *p=*.03; children overlapping < non-overlapping: *z*=2.56, *p*=.01; adults no significant difference between overlapping and non-overlapping: *z=*-0.58, *p*=.56).
