# Supplementary material for: Related memories exhibit interference during learning in adults but later retrieval in children
Source: PLoS One. 2026 Sep 11;21(9):e0356464. doi: 10.1371/journal.pone.0356464 (PMC13567822; doi:10.1371/journal.pone.0356464)
Supplement: Supplementary file 3 [file pone.0356464.s002.docx]

## Supporting Tables

**Table S1. Self-identified ethnicity for included participants.**

| Ethnicity | Percentage of participant sample |
| --- | --- |
| White | 29.1% |
| Chose not to specify | 19.5% |
| Mixed or identified with multiple ethnic groups | 14.5% |
| Chinese | 16.4% |
| Filipino | 7.7% |
| South Asian | 6.4% |
| Black | 1.8% |
| Arab | 0.9% |
| Latin American | 0.9% |
| Persian | 0.9% |
| Japanese | 0.5% |
| Metis | 0.5% |
| Slavic | 0.5% |
| West Asian | 0.5% |

**Table S2. Duration of each experimental phase (Phases I-III) and delays between phases by participant age group.**

| Age group |  | Phase I | Phase II | Phase III | I-II Delay^2^ | II-III Delay^3^ |
| --- | --- | --- | --- | --- | --- | --- |
| Children | *Median (SE)* | 5:00 (0:10)^1^ | 3:24 (0:10) | 1:06 (0:01) | 0:48 (0:02) | 0:06 (0:00.3) |
|  | *Range* | 1:36-13:30 | 1:36-13:30 | 0:48-2:12 | 0:00-3:30 | 0:00-0:12 |
| Adults | *Median (SE)* | 7:30 (0:35) | 3:48 (0:09) | 1:12 (0:01) | 1:12 (0:05) | 0:06 (0:00.5) |
|  | *Range* | 3:42-30:12 | 2:06-8:36 | 0:54-1:48 | 0:42-5:12 | 0:00-0:12 |

^1^Durations expressed in minutes:seconds format

^2^Duration of delays between completion of phase I and beginning of phase II (I-II Delay) and between completion of phase II and beginning of phase III (II-III Delay). Delays were calculated as the difference between timestamps saved at the end of the previous phase, i.e., immediately after the participant reached the learning criterion for the second map in the phase, and the beginning of the next phase, i.e., the first frame of the animation depicting the monster moving to the next map. Delays of 0 seconds indicate instances in which the participant chose to progress immediately to the next phase and instructions were given while the monster animation played. Monster animations were between 4 and 8 seconds.

## Supporting Figures

**Fig S1.** **Child performance on new learning and memory test phases according to age**. (**a**) Learning rate in new learning phase for children according to map overlap and child age. (**b**) Accuracy on final memory test for children according to map overlap and child age. Smaller dots represent individual participant values which are coloured according to condition (non-overlapping in blue [left] and pink [right]; overlapping in yellow). Lines represent model predictions and ribbons represent 95% confidence intervals. Histograms at top indicate number of children at each age (binned by age in years) included in the sample.
