# Supplementary material for: Related memories exhibit interference during learning in adults but later retrieval in children
Source: PLoS One. 2026 Sep 11;21(9):e0356464. doi: 10.1371/journal.pone.0356464 (PMC13567822; doi:10.1371/journal.pone.0356464)
Supplement: Peer review history

# Peer review: Decision Letter 0

**DOI:** 10.1371/journal.pone.0356464.r001

**Authors:** Bradley King

10 Apr 2026

Dear Dr. Woodbury,

plosone@plos.org. When you're ready to submit your revision, log on to https://www.editorialmanager.com/pone/ and select the 'Submissions Needing Revision' folder to locate your manuscript file.

- A letter that responds to each point raised by the academic editor and reviewer(s). You should upload this letter as a separate file labeled 'Response to Reviewers'.
- A marked-up copy of your manuscript that highlights changes made to the original version. You should upload this as a separate file labeled 'Revised Manuscript with Track Changes'.
- An unmarked version of your revised paper without tracked changes. You should upload this as a separate file labeled 'Manuscript'.

If you would like to make changes to your financial disclosure, please include your updated statement in your cover letter. Guidelines for resubmitting your figure files are available below the reviewer comments at the end of this letter.

If applicable, we recommend that you deposit your laboratory protocols in protocols.io to enhance the reproducibility of your results. Protocols.io assigns your protocol its own identifier (DOI) so that it can be cited independently in the future. For instructions see: https://journals.plos.org/plosone/s/submission-guidelines#loc-laboratory-protocols. Additionally, PLOS ONE offers an option for publishing peer-reviewed Lab Protocol articles, which describe protocols hosted on protocols.io. Read more information on sharing protocols at https://plos.org/protocols?utm_medium=editorial-email&utm_source=authorletters&utm_campaign=protocols.

As the corresponding author, your ORCID iD is verified in the submission system and will appear in the published article. PLOS supports the use of ORCID, and we encourage all coauthors to register for an ORCID iD and use it as well. Please encourage your coauthors to verify their ORCID iD within the submission system before final acceptance, as unverified ORCID iDs will not appear in the published article. *Only*  the individual author can complete the verification step; PLOS staff *cannot*  verify ORCID iDs on behalf of authors.

We look forward to receiving your revised manuscript.

Kind regards,

Bradley R. King

Academic Editor

PLOS One

Journal Requirements:

When submitting your revision, we need you to address these additional requirements.

1. Please ensure that your manuscript meets PLOS ONE's style requirements, including those for file naming. The PLOS ONE style templates can be found at

https://journals.plos.org/plosone/s/file?id=wjVg/PLOSOne_formatting_sample_main_body.pdf and

https://journals.plos.org/plosone/s/file?id=ba62/PLOSOne_formatting_sample_title_authors_affiliations.pdf

2. Thank you for stating the following in the Acknowledgments Section of your manuscript:

“This research was supported by Natural Sciences and Engineering Research Council of Canada (NSERC) Discovery Grants (RGPIN-2018-04933, RGPIN-2025-06189) and Discovery Launch Supplement (DGECR-2018-00252), the Canada Foundation for Innovation John R. Evans Leaders Fund (36876), the Ontario Research Fund, and University of Toronto startup funds all awarded to MLS as well as the University of Toronto Excellence Award in Natural Sciences & Engineering to TY and the Ontario Graduate Scholarship to MW.”

We note that you have provided funding information that is not currently declared in your Funding Statement. However, funding information should not appear in the Acknowledgments section or other areas of your manuscript. We will only publish funding information present in the Funding Statement section of the online submission form.

Please remove any funding-related text from the manuscript and let us know how you would like to update your Funding Statement. Currently, your Funding Statement reads as follows:

“This research was supported by Natural Sciences and Engineering Research Council of Canada (NSERC) Discovery Grants (RGPIN-2018-04933, RGPIN-2025-06189) and Discovery Launch Supplement (DGECR-2018-00252) (https://nserc-crsng.canada.ca/en), the Canada Foundation for Innovation John R. Evans Leaders Fund (36876) (https://www.innovation.ca/), the Ontario Research Fund (https://www.ontario.ca/page/ontario-research-fund), and University of Toronto (https://www.utoronto.ca/) startup funds all awarded to MLS as well as the University of Toronto (https://www.utoronto.ca/) Excellence Award in Natural Sciences & Engineering to TY and Ontario Graduate Scholarship to MW. The funders played no role in study design, data collection and analysis, decision to publish, or preparation of the manuscript.”

Please include your amended statements within your cover letter; we will change the online submission form on your behalf.

3. Thank you for stating the following financial disclosure:

“This research was supported by Natural Sciences and Engineering Research Council of Canada (NSERC) Discovery Grants (RGPIN-2018-04933, RGPIN-2025-06189) and Discovery Launch Supplement (DGECR-2018-00252) (https://nserc-crsng.canada.ca/en), the Canada Foundation for Innovation John R. Evans Leaders Fund (36876) (https://www.innovation.ca/), the Ontario Research Fund (https://www.ontario.ca/page/ontario-research-fund), and University of Toronto (https://www.utoronto.ca/) startup funds all awarded to MLS as well as the University of Toronto (https://www.utoronto.ca/) Excellence Award in Natural Sciences & Engineering to TY and Ontario Graduate Scholarship to MW. The funders played no role in study design, data collection and analysis, decision to publish, or preparation of the manuscript.”

We note that one or more of the authors is affiliated with the funding organization, indicating the funder may have had some role in the design, data collection, analysis or preparation of your manuscript for publication; in other words, the funder played an indirect role through the participation of the co-authors. If the funding organization did not play a role in the study design, data collection and analysis, decision to publish, or preparation of the manuscript and only provided financial support in the form of authors' salaries and/or research materials, please do the following:

1. Review your statements relating to the author contributions, and ensure you have specifically and accurately indicated the role(s) that these authors had in your study. These amendments should be made in the online form.

2. Confirm in your cover letter that you agree with the following statement, and we will change the online submission form on your behalf:

“The funder provided support in the form of salaries for authors [insert relevant initials], but did not have any additional role in the study design, data collection and analysis, decision to publish, or preparation of the manuscript. The specific roles of these authors are articulated in the ‘author contributions’ section.

4. Please note that your Data Availability Statement is currently missing the DOI/accession number of each dataset OR a direct link to access each database. If your manuscript is accepted for publication, you will be asked to provide these details on a very short timeline. We therefore suggest that you provide this information now, though we will not hold up the peer review process if you are unable.

5. Your ethics statement should only appear in the Methods section of your manuscript. If your ethics statement is written in any section besides the Methods, please delete it from any other section.

6. We note that Figure 2 in your submission contain copyrighted images. All PLOS content is published under the Creative Commons Attribution License (CC BY 4.0), which means that the manuscript, images, and Supporting Information files will be freely available online, and any third party is permitted to access, download, copy, distribute, and use these materials in any way, even commercially, with proper attribution. For more information, see our copyright guidelines: http://journals.plos.org/plosone/s/licenses-and-copyright.

We require you to either (1) present written permission from the copyright holder to publish these figures specifically under the CC BY 4.0 license, or (2) remove the figures from your submission:

1. You may seek permission from the original copyright holder of Figure(s) [#] to publish the content specifically under the CC BY 4.0 license.

We recommend that you contact the original copyright holder with the Content Permission Form (http://journals.plos.org/plosone/s/file?id=7c09/content-permission-form.pdf) and the following text:

“I request permission for the open-access journal PLOS ONE to publish XXX under the Creative Commons Attribution License (CCAL) CC BY 4.0 (http://creativecommons.org/licenses/by/4.0/). Please be aware that this license allows unrestricted use and distribution, even commercially, by third parties. Please reply and provide explicit written permission to publish XXX under a CC BY license and complete the attached form.”

Please upload the completed Content Permission Form or other proof of granted permissions as an "Other" file with your submission.

In the figure caption of the copyrighted figure, please include the following text: “Reprinted from [ref] under a CC BY license, with permission from [name of publisher], original copyright [original copyright year].”

2. If you are unable to obtain permission from the original copyright holder to publish these figures under the CC BY 4.0 license or if the copyright holder’s requirements are incompatible with the CC BY 4.0 license, please either i) remove the figure or ii) supply a replacement figure that complies with the CC BY 4.0 license. Please check copyright information on all replacement figures and update the figure caption with source information. If applicable, please specify in the figure caption text when a figure is similar but not identical to the original image and is therefore for illustrative purposes only.

7. We notice that your supplementary figures are uploaded with the file type 'Figure'. Please amend the file type to 'Supporting Information'. Please ensure that each Supporting Information file has a legend listed in the manuscript after the references list.

8. Please include captions for your Supporting Information files at the end of your manuscript, and update any in-text citations to match accordingly. Please see our Supporting Information guidelines for more information: http://journals.plos.org/plosone/s/supporting-information.

9. If the reviewer comments include a recommendation to cite specific previously published works, please review and evaluate these publications to determine whether they are relevant and should be cited. There is no requirement to cite these works unless the editor has indicated otherwise.

[Note: HTML markup is below. Please do not edit.]

Reviewers' comments:

Reviewer's Responses to Questions

**Comments to the Author**

1. Is the manuscript technically sound, and do the data support the conclusions?

Reviewer #1: Yes

Reviewer #2: Yes

**********

2. Has the statistical analysis been performed appropriately and rigorously?

Reviewer #1: Yes

Reviewer #2: Yes

**********

3. Have the authors made all data underlying the findings in their manuscript fully available?

The PLOS Data policy

Reviewer #1: Yes

Reviewer #2: No

**********

4. Is the manuscript presented in an intelligible fashion and written in standard English?

Reviewer #1: Yes

Reviewer #2: Yes

**********

Reviewer #1: The authors investigated whether children and adults differ in how they: (1) learn novel object-location associations embedded into a previously encountered context, and (2) recall the object-location associations initially learned in that context. The results of this behavioral study suggest that, while children learn novel information at a similar rate independently of whether it is presented in a new or previously encountered context, the latter results in a slower learning rate in adults. Conversely, children’s memory performance was significantly lower when the retrieved initial object-location associations were presented in a context in which novel overlapping associations had later been encoded. The authors interpret these findings to suggest that adults display interference during learning, while children at retrieval.

The pre-registration of the study and (anticipated) open availability of data are commendable, and the statistical analyses are sound and well-motivated. Nevertheless, I have a few comments.

My first major comment is about the timing of the experimental phases. The study paradigm is inspired by previous work which demonstrated learning facilitation of new object-location associations when inserted in a schema-compatible framework; however, unlike previous work, this paradigm does not allow ample consolidation time between the acquisition of the schema memories and that of the schema-compatible ones. The authors briefly acknowledge this as a limitation in the Discussion, but in my opinion the short delays need to be more clearly pointed out throughout the entire text, and the claims that what is being tested is the effect of “established prior knowledge” on new learning toned down, as it is unclear whether these un-consolidated memories can actually be leveraged as prior knowledge. For example, I would ask to tone down the sentence at lines 113-116: “We ensured that initial map memories were well-established, and thus could serve as a scaffold for new learning, by repeatedly exposing participants to these object-location associations during a preceding learning phase until they remembered every association with perfect accuracy”, as I am not convinced that perfect accuracy after a short learning session ensures well-establishment of a memory and its ready access for scaffolding new learning.

In short, I would like the authors to:

1. Point out and motivate their choice of deviating from previous related work by allowing only short delays between experimental phases in the Introduction and/or Methods of the manuscript, instead of leaving this important note only in the Discussion

2. Add details in the Methods regarding the length of both the learning phases and the delays between them. How long was each learning phase on average, and what was the variance within each group? Was there a set delay between learning phases I and II, and II and III? If not, what was the average delay?

3. Investigate in an additional analysis whether the length of phase I to phase II delay correlated with subsequent rate of learning in the overlapping environment

4. Discuss alternative explanations for the observed results that do not assume the initially learned map memories were consolidated and able to act as scaffold for the novel ones. For example, is it possible that the learning deficit observed in adults during phase II may reflect taxation of short-term memory capacity during learning, if such learning was being grouped by map (considering there are twice the number of items to encode in the overlapping map)?

The manuscript is also missing details on a few other points. The introduction is overall clear; however, it is too vague in some places. For example, the paragraph at lines 88-95 states: “existing work has indexed neurobiological markers reflecting the extent to which prior memories are leveraged during learning” – I would like to authors to please explicitly state which markers they are referring to. Likewise, at line 95: “using behavioural measures of acquisition akin to past adult work” – such as? Please explicitly define these measures. And again, at lines 108-111: “[we used] a spatial learning paradigm which was inspired by influential animal work and mirrors approaches that have proven fruitful in human adult research” – brief descriptions of these paradigms are needed, so that the reader can compare them to the one presented in the current study (especially regarding timing, as mentioned above).

Finally, tying into my earlier comment to please specify the “neurobiological markers reflecting the extent to which prior memories are leveraged during learning”, I would appreciate some more speculation in the Discussion as to what the behavioral results of this study may imply regarding the differences in neurobiological mechanisms underlying memory-memory interactions in children vs adults (currently very briefly touched upon at lines 346-348).

Minor comments:

Line 72 – the term “initial memories” is somewhat unclear; consider using “initially learned” (also in the rest of the manuscript)

Line 74 – The phrasing of “while adults can bring to mind their prior memories such that it shapes new learning” is awkward, I would suggest simplifying to: “while adults can bring to mind their prior memories to shape new learning”

Line 76 – please insert a comma before “respectively”

Line 79 – the sentence structure suggests that the studies referenced here were all conducted in adult humans, but a work conducted in rodents is cited. Please rephrase to make this clear.

Line 81 – for consistency with earlier sections of the Introduction, I suggest using “facilitate” rather than “ground”

Line 97 – I find “to recall what happened where and when” too colloquial, I suggest using “to recall contextual details”. Please similarly update line 363.

Line 228 – I would add “subsequent” learning

For consistency with the Methods, in the Results please explicitly refer to the learning phases corresponding to each presented test (e.g., lines 229-232: phase I; lines 232-236, etc.).

Line 243 – please change “confirmed” to “suggested”

Line 282 – please change again “confirmed our results…” to “investigated whether our results…”

Lines 297-299 – in this otherwise very solid paper statistics-wise, I would prefer not to see results reported as “marginally significant”. Please change this to (something along the lines of): “Children tended to display higher interference during memory test, although this difference was not significant”. Please also add the caveat that the difference was not significant to line 377 in the Discussion.

Lines 395-397 – as this experiment is purely behavioural, stating that “we demonstrate key differences in how children and adults build knowledge” is, in my opinion, too strong a claim. Change to (something along the lines of): “these results suggest there may be key differences in how children and adults build knowledge”

Supplementary Methods – please put the list of ethnicities in a table instead of text, and in descending order from most to least represented ethnicity.

Supplement – page 5, typo: “there was still in interaction”

Reviewer #2: This manuscript describes a behavioral experiment with adults (n=59) and children (n=161, 4-16 years) that investigates interactions among related memories during learning and retrieval. Participants memorized the identities of each card within different maps. One map background was repeated to create potential interference. The number of cards within the map was larger for adults compared to child to equate difficulty. Learning rates were the same for children and adults when learning the non-overlapping maps. However, adults' learning was more sensitive to the overlap manipulation than children’s: adults learned the overlapping map more slowly than the non-overlapping maps, whereas children showed equivalent learning rates in both conditions. By contrast, for memory retrieval, children’s memory was more sensitive to overlap than adults. Children’s memory accuracy was lower for the overlapping map compared to the non-overlapping map, but adults’ memory did not differ between the maps. Overall, I enjoyed reading this manuscript. I think the experimental design is a clever and appropriate way of targeting the research questions, the writing is clear, and figures provide useful visual aids. I have only a few questions about interpreting the results:

1) Given the age-group differences between children and adults regarding the overlap manipulation for learning and retrieval and the large age range tested in children, it is somewhat surprising that the effect of overlap did not vary as a function of age within children. How do you interpret these seemingly conflicting findings? Do you think it is an issue of statistical power within the child group given the dense sampling between 6-9 years? Potentially there is an inflection point after age 16 rather than continuous change?

2) Figure 2A seems to show a striking bimodal distribution of learning rates in both children and adults, where there is a larger cluster of slower learners and a smaller but still large cluster of much faster learners. How do you interpret these different clusters in terms of potentially different learning strategies? I see there is an exploratory analysis relating interference during learning and interference during memory, which found no relation, but do these different clusters of learning speed relate to later memory performance?

**********

what does this mean?). If published, this will include your full peer review and any attached files.

If you choose “no”, your identity will remain anonymous but your review may still be made public.

**Do you want your identity to be public for this peer review?**  For information about this choice, including consent withdrawal, please see our Privacy Policy

Reviewer #1: No

Reviewer #2: No

**********

[NOTE: If reviewer comments were submitted as an attachment file, they will be attached to this email and accessible via the submission site. Please log into your account, locate the manuscript record, and check for the action link "View Attachments". If this link does not appear, there are no attachment files.]

To ensure your figures meet our technical requirements, please review our figure guidelines: https://journals.plos.org/plosone/s/figures

You may also use PLOS’s free figure tool, NAAS, to help you prepare publication quality figures: https://journals.plos.org/plosone/s/figures#loc-tools-for-figure-preparation.

NAAS will assess whether your figures meet our technical requirements by comparing each figure against our figure specifications.

# Author comment: Author response to Decision Letter 1

**DOI:** 10.1371/journal.pone.0356464.r002

15 Jul 2026

Authors’ Response

Our sincerest thank you to the Reviewers and Editor for their time and thoughtful consideration of our manuscript. The Reviewers found our manuscript statistically rigorous and the study carefully designed; however, they had a few suggestions for improvement which we have revised the manuscript to address as detailed in this letter. For example, Editor and Reviewer comments prompted us to reassess and adjust our use of Bayesian statistics; better motivate and clarify details of our single-day learning paradigm, include additional detail in our Introduction; and highlight additional outstanding questions regarding strategy use and developmental differences into adolescence in our Discussion. We thank the Reviewers and Editor again for their suggestions which we believe have greatly strengthened the manuscript. Below, Editor and Reviewer comments are in italics and our responses are in normal font.

EDITOR

Thank you for submitting your manuscript to PLOS ONE. After careful consideration, we feel that it has merit but does not fully meet PLOS ONE’s publication criteria as it currently stands. Therefore, we invite you to submit a revised version of the manuscript that addresses the points raised during the review process.

The comments from the the reviewers are listed below. I agree with the reviewers that addressing these inquiries would result in an improved manuscript.

Thank you for your time and thoughtful feedback, as well as the opportunity to submit a revision. We agree that addressing Reviewer feedback has strengthened the manuscript.

In addition to their comments, I kindly request that you address the following issues/questions I had while reading:

a) small detail but the minimum age of the children is sometimes listed as 4 (e.g., abstract) but other times listed as 3 (e.g., when discussing median split; and Figure S1 looks like youngest children are less than 4). Please clarify and be consistent throughout the text.

Thank you for your careful eye and for noting this error. The actual minimum age in our sample was 3.9 years old, and so when 4 years old was mentioned this was a typo. We have corrected this error throughout the paper (p. 6 and 10).

With respect to Figure S1, we understand the Editor’s point that as dots in the originally submitted figure were to the left of the 4 year old tick mark on the x-axis, it appeared as though the age range extended substantially below 4 years. This was merely an artifact of our plotting approach, in which points were “dodged” in ggplot (i.e., offset slightly left and right) to separate the non-overlapping condition in blue/pink from the non-overlapping condition in yellow. Upon reflection and consideration of this comment, we realized that our approach in the original submission of analyzing age as integers could be improved upon by instead computing a more precise age including decimal places. For the purposes of this revision, we have revised our approach and re-made this figure to reflect these more precise ages. In so doing, we have dropped the “dodging” approach in the plotting such that, related to the Editor’s point, now participants’ points are exactly aligned with their age on the x-axis. A helpful side effect if this change is that readers can better visualize the within-participant differences (i.e., as now participant ages are more likely to be unique and therefore dots appearing at the same point along the x-axis are likely from the same participant).

We have repeated all analyses using these more precise ages. While this does not have any effect on our primary analyses which considered the whole group of participants irrespective of age, it did affect our reporting of age range, mean, and standard deviation (p. 6) as well as our median split (p. 10; results on p. 11-13). This change did not have a meaningful impact on any of the results or conclusions, and we have updated the particular statistics throughout the manuscript to reflect the results from analyses using precise ages. (e.g., Supporting Results in S1 File p. 2 and 4).

Thank you for bringing this to our attention and prompting this shift in our consideration of age as a more precise value. We believe this change both improves the work overall and helps to clarify how our age range maps onto the graph in Figure S1.

b) I appreciate the use of Bayesian statistics to complement the frequentist stats that served as the primary approach, but was confused as to why these were ONLY done for null results. Bayes Factors can certainly be used to provide the strength of of the evidence in favor of the alternative model (as compared to null) and readers will benefit from having this information. Related, Bayes Factors can be provided for the significant interaction terms that serve as the primary effects reported in this manuscript and readers will benefit from being provided the strength of this evidence. I kindly request a more thorough reporting of these BFs.

Thank you for this thoughtful suggestion for how to strengthen our results reporting and increase the consistency of our statistical approach. This comment prompted us to think more deeply about how our preregistered frequentist analyses and potential Bayesian follow-ups ought to be combined. Having considered extant perspectives on this issue (Dienes, 2024 JEP: Human perception & performance; Schreiner & Kunde, 2025 JEP: General), we have arrived at the conclusion that it is more appropriate to solely report our planned frequentist statistics rather than additionally include either partial or comprehensive Bayesian testing.

Our reasoning for this decision is as follows: While other research (e.g., Liu et al., 2023, Cognition; Qiao et al., 2023, Current Psychology; Tsuji & Imaizumi, 2022, Scientific Reports) has adopted a similar approach to the one we employed in our initial submission, in which Bayes Factors are reported solely for non-significant frequentists effects in order to support stronger inferences for null effects, recent work argues that this practice can be problematic (Dienes, 2024; Schreiner & Kunde, 2025). Specifically, Dienes (2024) and Schreiner & Kunde (2025) argue that this approach results in unequal treatment of significant and non-significant effects because they are tested with different assumptions and constraints: While significant effects are subject to frequentist assumptions alone, non-significant effects are additionally subject to those associated with Bayesian tests. This can lead to issues. For example, when non-significant frequentist effects are followed up by a Bayesian test with uninformative priors, there may be a bias to yield evidence in favour of the null because such Bayesian tests have a tendency to produce false negatives (Schreiner & Kunde, 2025). By contrast, the same false negative bias would not affect results that were significant under the frequentist approach, as no Bayesian follow-ups were conducted. Given the risks of this uneven statistical approach, both Schreiner & Kunde (2025) and Dienes (2024) argue that one should use the same inferential system consistently in reporting the results, regardless of whether a frequentist or Bayesian approach is selected.

As for why we have opted to forgo Bayes Factor reporting altogether rather than incorporating Bayesian statistics more thoroughly, we believe it is most appropriate to follow our preregistered frequentist approach which was planned to mirror previous related work in adults (Guo & Yang, 2019; Sommer, 2017; van Buuren et al., 2014). Dual reporting of statistics from multiple inferential systems runs the risk of producing conflicting findings (Dienes, 2024). Instead, the most straightforward and appropriate course of action is to follow the originally planned frequentist analyses. Consequently, we have removed Bayes Factor reporting for null effects wherever previously included in the Results (Fig 1 and p. 10, 11) and Supporting Results (S1 File p. 3) along with the description of these analyses in the Methods (p. 10). Further, as frequentist statistical tests do not allow for the same interpretative strength for null effects as Bayes Factors, we have adjusted our discussion of null effects accordingly throughout the Results and Discussion (e.g., p. 14 and 15).

We thank you again for pointing out the uneven reporting of Bayesian statistics in our results. Your comment led us to consider potential pitfalls of our previous approach, ultimately allowing us better align our statistical reporting with contemporary recommendations for the field.

Review Comments to the Author

REVIEWER #1

The authors investigated whether children and adults differ in how they: (1) learn novel object-location associations embedded into a previously encountered context, and (2) recall the object-location associations initially learned in that context. The results of this behavioral study suggest that, while children learn novel information at a similar rate independently of whether it is presented in a new or previously encountered context, the latter results in a slower learning rate in adults. Conversely, children’s memory performance was significantly lower when the retrieved initial object-location associations were presented in a context in which novel overlapping associations had later been encoded. The authors interpret these findings to suggest that adults display interference during learning, while children at retrieval.

The pre-registration of the study and (anticipated) open availability of data are commendable, and the statistical analyses are sound and well-motivated. Nevertheless, I have a few comments.

Thank you for your time and feedback. We address each of your comments below.

R1.1) My first major comment is about the timing of the experimental phases. The study paradigm is inspired by previous work which demonstrated learning facilitation of new object-location associations when inserted in a schema-compatible framework; however, unlike previous work, this paradigm does not allow ample consolidation time between the acquisition of the schema memories and that of the schema-compatible ones. The authors briefly acknowledge this as a limitation in the Discussion, but in my opinion the short delays need to be more clearly pointed out throughout the entire text, and the claims that what is being tested is the effect of “established prior knowledge” on new learning toned down, as it is unclear whether these un-consolidated memories can actually be leveraged as prior knowledge. For example, I would ask to tone down the sentence at lines 113-116: “We ensured that initial map memories were well-established, and thus could serve as a scaffold for new learning, by repeatedly exposing participants to these object-location associations during a preceding learning phase until they remembered every association with perfect accuracy”, as I am not convinced that perfect accuracy after a short learning session ensures well-establishment of a memory and its ready access for scaffolding new learning.

Thank you for highlighting this important theoretical and methodological difference between our work and previous related studies. We agree that the reduced time between initial and new learning in our paradigm compared to those used in prior related work (e.g., van Buuren et al., 2014; Sommer, 2017) may have led to interference rather than facilitation in adults in part because initial memories were not consolidated prior to new learning. We included mention of this account in the original Discussion and retain it in the revision (p. 15). Importantly, we wish to clarify that by referring to initially formed memories as “established” we did not mean to imply that they were consolidated. Rather, we were referencing that memories of the initial locations in the overlapping condition were likely relatively strong given participants had learned them perfectly by the end of Phase I. However, we take the Reviewer’s point that referring to these memories as “established” may create the impression that these memories must be strong, and perhaps even consolidated. We have therefore made changes to the way we describe these initial memories, discussed further below.

We also wish to highlight that there is a substantial body of literature suggesting that even recent, unconsolidated memories can be leveraged during the acquisition of new knowledge. While the schema literature indeed primarily focuses on the influence of remote, consolidated memories, other work has found that even on the same day, manipulations affecting the likelihood of reactivating related memories during subsequent learning can influence how new information is encoded (e.g., Molitor et al., 2021; Richter et al., 2016; Schlichting et al., 2015; Zeithamova et al., 2012) with either positive (Anderson & McCulloch, 1999; Schlichting et al., 2014) or negative (Bramao et al., 2025; Oren & Ash, 2017) effects. For instance, Zeithamova et al. (2012) found that when learning new associations, adults showed neural evidence of reactivating related pairs that had been acquired immediately beforehand. Moreover, the degree to which initial memories were reactivated during learning predicted how strongly new memories were integrated into initial ones. Such online integration is thought to underpin the benefit of prior memories for learning and/or later memory for new, related information (van Kesteren et al., 2012, Trends in Neurosciences; Zeithamova et al. 2012, Frontiers in Human Neuroscience). Further, some work finds that new learning is integrated with memories formed on the same day to a similar (Fang et al., 2023) or even greater (Zeithamova & Preston, 2017) extent than those formed on a different day. It was this work that motivated our prediction that even on a shorter timescale, adults would access recent memories during new learning and thus demonstrate facilitation. Notably, however, even if learning can be influenced by recently formed (same-day) memories as seen in prior work, it is nevertheless possible that consolidation could still shift the nature of these memory-memory interactions.

In context of this prior work, our reasons for having participants form initial and new memories within a single session rather than over multiple days were twofold. First, we aimed to empirically test whether facilitation effects observed in prior adult work using spatial learning paradigms (e.g., van Buuren et al., 2014) were also observed within a compressed timeframe, when initial and new memories were formed on the same day. We predicted that adults would call to mind and integrate initially learned spatial maps with new location memories, demonstrating speeded learning in the overlapping compared to non-overlapping condition as a result. Our results instead suggested that adults were impeded by their recently acquired memories. In the Discussion, we speculate as to why this may be the case (p. 15), including consideration of the shorter timeframe and reduced learning exposure to initial maps compared to prior similar work. Second, we had participants complete initial and new learning in a single session to ensure feasibility for collecting a large in-person developmental sample. The short completion time, typically less than 30 minutes, enabled us to collect data at community venues where many potential participants are only available for a short period.

We have revised our manuscript in a number of places (e.g., p. 5 and p. 15) to highlight the difference between this and prior work using similar spatial learning tasks more clearly as well as to explain our reasoning for this design choice. First, we mention the same-day nature of our experiment in our revised Abstract (p. 2) so that this aspect of the design is clear to the reader from the beginning. Second, we have changed the wording of the passage quoted by the Reviewer to remove the potential implication that initial memories must scaffold new learning and to rather emphasize that the task was designed with the aim to establish strong enough initial map memories such that they may interact with new learning, positively or negatively (p. 5). Third, we have included explicit description of how the timescale of our paradigm differs from

Attachment Submitted filename: response to reviewers.pdf

# Peer review: Decision Letter 1

**DOI:** 10.1371/journal.pone.0356464.r003

**Authors:** Bradley King

5 Aug 2026

Related memories exhibit interference during learning in adults but later retrieval in children

PONE-D-26-10616R1

Dear Dr. Woodbury,

We’re pleased to inform you that your manuscript has been judged scientifically suitable for publication and will be formally accepted for publication once it meets all outstanding technical requirements.

Within one week, you’ll receive an e-mail detailing the required amendments. When these have been addressed, you’ll receive a formal acceptance letter and your manuscript will be scheduled for publication.

An invoice will be generated when your article is formally accepted. Please note, if your institution has a publishing partnership with PLOS and your article meets the relevant criteria, all or part of your publication costs will be covered. Please make sure your user information is up-to-date by logging into Editorial Manager at Editorial Manager® and clicking the ‘Update My Information' link at the top of the page. For questions related to billing, please contact billing support.

If your institution or institutions have a press office, please notify them about your upcoming paper to help maximize its impact. If they’ll be preparing press materials, please inform our press team as soon as possible -- no later than 48 hours after receiving the formal acceptance. Your manuscript will remain under strict press embargo until 2 pm Eastern Time on the date of publication. For more information, please contact onepress@plos.org.

Kind regards,

Bradley R. King

Academic Editor

PLOS One

Additional Editor Comments (optional):

Reviewers' comments:

Reviewer's Responses to Questions

**Comments to the Author**

Reviewer #1: All comments have been addressed

Reviewer #2: All comments have been addressed

**********

2. Is the manuscript technically sound, and do the data support the conclusions?

Reviewer #1: Yes

Reviewer #2: (No Response)

**********

3. Has the statistical analysis been performed appropriately and rigorously?

Reviewer #1: Yes

Reviewer #2: (No Response)

**********

4. Have the authors made all data underlying the findings in their manuscript fully available?

The PLOS Data policy

Reviewer #1: Yes

Reviewer #2: (No Response)

**********

5. Is the manuscript presented in an intelligible fashion and written in standard English?

Reviewer #1: Yes

Reviewer #2: (No Response)

**********

Reviewer #1: The authors have thoroughly explained their reasoning regarding experimental design choices, improved text clarity, demonstrated robustness of findings with control analyses, and in sum they have satisfactorily addressed all of my comments. I am happy to recommend the current manuscript for publication.

Reviewer #2: I appreciate the authors' thorough responses to the points raised by myself and the other reviewer and the resulting revisions to the manuscript

**********

what does this mean?). If published, this will include your full peer review and any attached files.

If you choose “no”, your identity will remain anonymous but your review may still be made public.

**Do you want your identity to be public for this peer review?**  For information about this choice, including consent withdrawal, please see our Privacy Policy

Reviewer #1: No

Reviewer #2: No

**********

# Editor report: Acceptance letter

**DOI:** 10.1371/journal.pone.0356464.r004

**Authors:** Bradley King

PONE-D-26-10616R1

PLOS One

Dear Dr. Woodbury,

I'm pleased to inform you that your manuscript has been deemed suitable for publication in PLOS One. Congratulations! Your manuscript is now being handed over to our production team.

At this stage, our production department will prepare your paper for publication. This includes ensuring the following:

* All references, tables, and figures are properly cited

* All relevant supporting information is included in the manuscript submission,

* There are no issues that prevent the paper from being properly typeset

You will receive further instructions from the production team, including instructions on how to review your proof when it is ready. Please keep in mind that we are working through a large volume of accepted articles, so please give us a few days to review your paper and let you know the next and final steps.

Lastly, if your institution or institutions have a press office, please let them know about your upcoming paper now to help maximize its impact. If they'll be preparing press materials, please inform our press team within the next 48 hours. Your manuscript will remain under strict press embargo until 2 pm Eastern Time on the date of publication. For more information, please contact onepress@plos.org.

You will receive an invoice from PLOS for your publication fee after your manuscript has reached the completed accept phase. If you receive an email requesting payment before acceptance or for any other service, this may be a phishing scheme. Learn how to identify phishing emails and protect your accounts at https://explore.plos.org/phishing.

If we can help with anything else, please email us at customercare@plos.org.

Thank you for submitting your work to PLOS One and supporting open access.

Kind regards,

PLOS One Editorial Office Staff

on behalf of

Dr. Bradley R. King

Academic Editor

PLOS One
